# In Vitro Evaluation of a Phage Cocktail Controlling Infections with *Escherichia coli*

**DOI:** 10.3390/v12121470

**Published:** 2020-12-19

**Authors:** Imke H. E. Korf, Sophie Kittler, Anna Bierbrodt, Ruth Mengden, Christine Rohde, Manfred Rohde, Andrea Kroj, Tatiana Lehnherr, Angelika Fruth, Antje Flieger, Hansjörg Lehnherr, Johannes Wittmann

**Affiliations:** 1 Leibniz Institute DSMZ—German Collection of Microorganisms and Cell Cultures, Inhoffenstraße 7B, 38124 Braunschweig, Germany; Chr@dsmz.de (C.R.); johannes.wittmann@dsmz.de (J.W.); 2 Institute for Food Quality and Food Safety, University of Veterinary Medicine Hannover, Foundation, Bischofsholer Damm 15, 30173 Hannover, Germany; sophie.kittler@tiho-hannover.de; 3 FINK TEC GmbH, Oberster Kamp 23, 59069 Hamm, Germany; michael.fink@finktec.com; 4 Food Inspection, Animal Welfare and Veterinary Service of the Land of Bremen, Border Control Post Bremerhaven, Senator-Borttscheller-Straße 8, 27568 Bremerhaven, Germany; ruth.mengden@lmtvet.bremen.de; 5 Central Facility for Microscopy, Helmholtz-Centre for Infection Research (HZI), Inhoffenstraße 7, 38124 Braunschweig, Germany; manfred.rohde@helmholtz-hzi.de; 6 PTC Phage Technology Center GmbH, Siemensstraße 42, 59199 Bönen, Germany; a.kroj@ptc-phage.com (A.K.); t.lehnherr@ptc-phage.com (T.L.); h.lehnherr@ptc-phage.com (H.L.); 7 Robert Koch Institute, Burgstraße 37, 38855 Wernigerode, Germany; frutha@rki.de (A.F.); fliegera@rki.de (A.F.)

**Keywords:** *E. coli*, bacteriophages, phage cocktail, colibacillosis, multidrug-resistant bacteria, biofilm, phage-resistant variants, APEC

## Abstract

Worldwide, poultry industry suffers from infections caused by avian pathogenic *Escherichia coli*. Therapeutic failure due to resistant bacteria is of increasing concern and poses a threat to human and animal health. This causes a high demand to find alternatives to fight bacterial infections in animal farming. Bacteriophages are being especially considered for the control of multi-drug resistant bacteria due to their high specificity and lack of serious side effects. Therefore, the study aimed on characterizing phages and composing a phage cocktail suitable for the prevention of infections with *E. coli*. Six phages were isolated or selected from our collections and characterized individually and in combination with regard to host range, stability, reproduction, and efficacy in vitro. The cocktail consisting of six phages was able to inhibit formation of biofilms by some *E. coli* strains but not by all. Phage-resistant variants arose when bacterial cells were challenged with a single phage but not when challenged by a combination of four or six phages. Resistant variants arising showed changes in carbon metabolism and/or motility. Genomic comparison of wild type and phage-resistant mutant E28.G28R3 revealed a deletion of several genes putatively involved in phage adsorption and infection.

## 1. Introduction

*Escherichia coli* is among the most common pathogens in poultry and the causative agent of avian colibacillosis, which refers to localized or systemic infections, e.g., septicemia or air sac disease [1]. Avian pathogenic *Escherichia coli* (APEC) can asymptomatically colonize chicken intestines [2] and infections most likely occur by inhalation of contaminated dust particles, followed by the development of clinical signs [1]. Colibacillosis results in decreased production and/or increased morbidity and mortality of the animals [1,3]. As a frequent disease it causes significant economic losses in poultry industry [4]. If bacterial infections occur, they are treated with antibiotics, which in turn promote the selection of multidrug resistant (MDR) bacteria. Caused by this selection and the accumulation of antibiotics in the environment, an increasing incidence of MDR bacteria endangers not only animal health, but also human health by the potential zoonotic transfer of MDR bacteria, e.g., through contaminated food [5,6]. APEC strains may additionally serve as reservoir for virulence-associated genes [7]. Reinforced by restrictions regarding the use of antibiotics in livestock farming, there is a high demand to develop new strategies to fight bacterial infections. During the last decade, bacteriophages (phages) have experienced a renaissance as alternatives to antibiotics for treating bacterial infections in humans and animals and for applications in food production [8,9,10]. They are abundant in intestinal and environmental ecosystems and offer several advantages like high specificity, self-limitation, and they lack side-effects when compared to antibiotics [11,12,13,14]. The body temperature of chickens is about 42 °C and phages will be exposed to microaerophilic/anaerobic conditions in the gut during treatment. However, in further processes of meat production, where phages can additionally be applied in regard to food safety, cooling conditions apply. Thus, phages suitable for application must be stable under these conditions. The use of more than one phage in a well composed phage cocktail can prevent the growth of phage resistant bacteria [10]. In recent years, several studies evaluated bacteriophage applications for combatting infections caused by avian pathogenic *E. coli* with different outcomes [15,16,17,18,19]. However, to our knowledge, none of these studies evaluated the use of a phage cocktail for preventing the introduction of potentially pathogenic *E. coli* into the flock. 

In accordance with the One-Health concept that connects human, animal, and environmental health and was a leading theme during the 2016 World Health Summit [20], this study aimed at isolating and characterizing phages for the prevention of *E. coli* infections in a model of gut-colonization in broilers in order to minimize the use of antibiotics, their accumulation in the environment and the selection of MDR bacteria. Six phages were selected and characterized regarding their host range, stability, and growth characteristics. Due to its outstanding efficiency, the phage G28 was also analyzed for its molecular characteristics. The efficacy of the single phages and two preparations combining four or six of them (four-phage cocktail: G28, TB49, AB27, EW2; six-phage cocktail: G28, TB49, TriM, AB27, KRA2, and EW2) was evaluated in vitro. Additionally, phage resistant isolates that occurred during in vitro experiments were analyzed. To prepare for subsequent in vivo experiments in broiler chickens, a model *E. coli* strain was identified, thoroughly characterized and selected for in vitro evaluation of the phages. This *E. coli* model strain E28 allows selective quantification and differentiation from other *E. coli* strains as a prerequisite for the evaluation of phage efficiency in a multi-strain environment in animal trials that were conducted in a separate study subsequent to our experiments. In this case, the phages were administered preventively via the drinking water [21]. 

## 2. Materials and Methods 

### 2.1. Bacterial Strains and Growth Conditions

All strains were routinely grown in lysogeny broth (LB) (2.5% Miller’s LB Broth Base™ powder (Invitrogen, Thermo Fisher Scientific, Waltham, MA, USA) or on agar plates of LB medium supplemented with 1.5% agar bacteriological No. 1 (*w*/*v*) (OXOID, Thermo Fisher Scientific, Waltham, MA, USA) overnight at 37 °C unless stated otherwise. ESBL-positive *E. coli* isolates from clinical material (DSM 101101-DSM 101142) around Hameln (Germany), ESBL-positive *E. coli* isolates from poultry skin of carcasses in France, Belgium, the Netherlands, and Germany (E07, E08, E17, E18, E28, E29, E37, E43, E50, E53), APEC (avian pathogenic *E. coli*) strains (DSM 103254-DSM 103266), strains from the *E. coli* collection of reference (ECOR) (ECOR10, 13, 17, 28, 47), an *E. coli* strain isolated from a pig farm (E64), and laboratory strains (K12 (DSM 498), DH5α (DSM 6897), MG1655 (DSM 18039), B (DSM 613)) were used for phage isolation, propagation and host range analyses. Clinical isolates and isolates from poultry skin belonged to different serotypes (see Table S1) and the APEC strains belonged to serotypes O1 (*n* = 4), O2 (*n* = 5) and O78 (*n* = 3). Serotyping of DSM 101101-DSM 101142, E07–E53, K12 and *E. coli* B was performed at the Robert Koch Institute (Wernigerode, Germany) according to Ørskov typing scheme. Serotype information of APEC strains were obtained by Prof. Marcus Fulde (FU Berlin) and for the ECOR-strains from [22,23]. *E. coli* isolates from poultry skin and 15 randomly selected clinical isolates (see Table S2) were further characterized by Alere Technologies GmbH (Jena, Germany) *E. coli* PanType [24] to ensure diversity of the strains used for host range analyses.

### 2.2. Phage Isolation, Purification, and Propagation

Phages were isolated from different manure and surface water samples, using five *E. coli* host strains between April 2008 and August 2016 (Table S3).

For phage isolation, enrichments were prepared from different samples and phages were detected by the double agar overlay technique as described by Kropinski et al. [25]. For detailed information see Supplemental Materials. 

### 2.3. Morphology of Phages and Analysis of Phage Bacteria Interactions

The morphology of phages was evaluated by transmission electron microscopy as previously described [26]. 

To study the adsorption of phage G28 (vB_EcoM-G28) on the surface of the *E. coli* wild type E28 and the mutant E28.G28R3 Field Emission Scanning Electron Microscopy (FESEM), and bacteriophage adsorption assays were performed as previously published [27,28]. For detailed information see Supplemental Materials. 

### 2.4. Host Range and Efficiency of Plating (EOP) Analysis

The phage host range was determined by spotting serial dilutions on double agar overlay plates containing 100 µL of a logarithmic culture of the potential host. After incubation for 14–16 h at 37 °C the plates were examined for lysis. The host was classified as sensitive, when single plaques could be detected. All combinations of phages and strains were tested at least twice in independent experiments. The analysis of the ability to form plaques at different incubation conditions was performed following previous published methods [29,30,31]. Briefly, double agar plates were prepared with E28 as host strain and incubated 1 h at 10, 20, 37, and 42 °C. Serial dilutions of phage lysates were spotted on the lawn and read after 18 h (37 °C, 42 °C), 42 h (20 °C) and 10 days (10 °C). For microaerophilic conditions, plates were incubated in an anaerobic jar using a candle at 37 °C for 18 h. The CO_2_ concentration reached by this method is about 3–5% (personal communication). The EOP was calculated using values of aerobic conditions at 37 °C as standard. A mean value was calculated from three independent experiments.

### 2.5. One-Step Growth Assays

One-step growth experiments were performed based on previously described protocols [32,33]. For detailed information see Supplemental Materials. 

### 2.6. Stability of Phages

To test the pH stability, phage lysates were diluted hundredfold in LB medium adjusted to pH values ranging from 1 to 12. After incubation for the indicated time at specified temperatures, phages were serially diluted in SM buffer. Viable phages were determined by soft-agar overlay assays, using E28 as host strain. Data are presented as mean of duplicate determinations. 

Stability during storage of bacteriophages was examined in PBS (2.7 mM KCl, 8 mM Na_2_HPO_4_, 1.7 mM NaH_2_PO_4_, pH 7.0), PBS supplemented with 0.1 or 5 g/L NaCl (all components from Carl Roth GmbH & Co KG, Karlsruhe, Germany), or LB medium as described above.

### 2.7. Identification of a Suitable E. coli Strain Serving as a Basis for In Vivo Experiments

A trackable strain for in vivo experiments was identified by the analysis of antibiotic resistance patterns and the Phenotypic MicroArray Gen III MicroPlates™ (Biolog, Hayward, CA, USA) as previously described [34]. The susceptibility of the *E. coli* strains E07, E08, E17, E18, E28, E29, E37, E43, E50, E53 towards antimicrobial substances (OXOID, Thermo Fisher Scientific, Waltham, MA, USA) was examined by agar disc diffusion based on German standard DIN 58940. Bacterial cultures were spread on Mueller–Hinton agar plates (OXOID, Thermo Fisher Scientific, Waltham, MA, USA) and antibiotic discs (Table S4) were placed onto dried plates. After incubation at 37 °C for 16 h, the diameter of the inhibition zones was measured. Susceptibility of ESBL-producing *E. coli* isolates to 36 different antibiotics was displayed as a heatmap using the heatmap.2 () function of the R gplots package [34]. For the Phenotypic MicroArray, preparation of bacterial suspensions and loading of the plates was performed according to the recommendations of the manufacturer. The plates were incubated in the OmniLog™ incubator (Biolog, Hayward, CA, USA) at 37 °C for 48 h. Every 15 min, the change of the redox dye values was automatically measured and recorded in order to determine the respiration curve kinetics. These were analyzed and visualized using the R package opm [35]. For each strain, one experiment was performed. Resistance to potassium tellurite and kanamycin was confirmed by growth control experiments of all strains on agar plates containing both substances.

### 2.8. Lytic Efficacy of Phages and Phage Preparations

E28 was grown overnight on LB agar plates at 37 °C. Bacteria were inoculated in LB medium and incubated at 37 °C with agitation at 123 rpm for 2 h. Cell numbers were determined using a Neubauer cell counter and adjusted to 2 × 10^8^ CFU/mL. Each well of a 96 well plate was filled with 160 µL LB medium and 20 µL cell suspensions. After 2 h, 20 µL phage lysates (2 × 10^8^ PFU/mL or serial dilutions) were added. Incubation was performed in a TECAN (Infinite^®^ 200 PRO, Männedorf, Switzerland) plate reader at 37 °C and 6 mm shaking amplitude. Every 15 min the absorbance at 600 nm was automatically measured for 24 h. LB medium alone and E28 without phages served as controls. Data are presented as mean of triplicate determinations of two independent experiments.

### 2.9. In Vitro Biofilm Model

The protocol for testing the efficacy of phage in *E. coli* biofilms was adjusted from Chibeu et al. [36] and Gu et al. [37]. For detailed information see Supplemental Materials. 

### 2.10. Isolation of Phage-Resistant Variants

Phage-resistant variants were isolated as previously reported [38] and their resistance patterns were further analyzed: Individual colonies of *E. coli* E28 were used to inoculate LB broth. The liquid cultures were incubated at 37 °C with agitation at 123 rpm for 2 h. Afterwards, cell numbers were determined and 100 μL of this culture were infected with single phages at MOI 100 to achieve complete lysis. After incubation for 10 min, the infected cells were mixed with 3 mL LB soft-agar (0.3%), plated on LB agar and incubated at 37 °C overnight. Then, 10–100 phage-resistant variants were obtained 24 h after treatment with TriM, TB49, EW2, KRA2 and over 1000 colonies were found for AB27. About 5 colonies of mutants after challenge with G28 were visible after a prolonged incubation time (48 h). Three to four single colonies were isolated from each plate and purified by 3–4 rounds of fractionated streaking. Phage resistance was confirmed by spot test. No phage-resistant variants could be isolated for phage AB27, but the isolates observed by this method had a strongly reduced EOP and were therefore analyzed.

### 2.11. Analysis of the Carbon Consumption of Phage-Resistant Variants Using the Biolog GN2 MicroPlateTM Assay

For the phenotypic MicroArray, preparation of bacterial suspensions and loading of the plates was performed according to the recommendations of the manufacturer. The plates were incubated in the OmniLog™ ID system (Biolog, Hayward, CA, USA) at 37 °C for 48 h. The color density was measured automatically every 15 min for 48 h. For each strain, two independent biological replicates were performed. The program pmm_kinetik.exe and the R package opm [35] were used for analysis. The substrates gentiobiose, D-psicose, turanose, formic acid, L-alaninamide, L-histidine, L-ornithine, L-threonine, and Tween 80 were excluded from the analyses, as no reproducible results could be observed.

### 2.12. Growth and Swimming Motility of Phage-Resistant Variants

Bacteria were grown overnight in LB medium, adjusted to an optical density (OD) at 600 nm of 0.01 in LB broth, and 200 µL were incubated in a 96 well plate in a TECAN (Infinite^®^ 200 PRO, Männedorf, Switzerland) plate reader at 37 °C and 6 mm shaking amplitude. Every 15 min the absorbance at 600 nm was automatically measured for 24 h. LB medium alone and E28 wild type served as controls. Data are presented as mean of triplicate determinations.

Media used to assay swimming motility of *E. coli* contained 2.5% Miller’s LB Broth Base™ powder and 0.3% (*w*/*v*) agar bacteriological No. 1 (Thermo Fisher Scientific, Waltham, MA, USA). After solidification of 9 mL/petri dish (55 mm, Greiner Bio-One, Kremsmünster, Austria), 1 µL of the corresponding overnight culture was placed in the middle of one plate. Plates were incubated at 37 °C for 18 h and the diameter of colonies was measured.

### 2.13. DNA Isolation from Phages and Bacteria

Phage DNA extraction based on phenol-chloroform extraction. For detailed information see Supplemental Materials. 

Isolation of bacterial DNA: DNA from *E. coli* was isolated using the GenElute™ Bacterial Genomic DNA Kit (Merck, Darmstadt, Germany) following the manufacturer’s instructions.

### 2.14. Library Preparation and Whole Genome Sequencing

Phage G28 was sequenced and assembled by Microsynth AG (Balgach, Switzerland) using Illumina technology (MiSeq (2 × 300 bp), Illumina, Inc., San Diego, CA, USA). Sequencing of E28 was already published [39]. Shearing of 150 ng gDNA of the mutant E28.G28R3 was performed in a microTUBE AFA Fiber Snap-Cap (Covaris, Inc., Woburn, MA, USA) with the Covaris^®^ S2 DNA shearing technology. To attain a fragment size of 300 bp, shearing was done at an intensity of 5, 10% duty cycle, 200 cycles per burst and a treatment time of 50 s. To prepare next-generation sequencing libraries the NEBNext Ultra™ kit (New England Biolabs Ltd., Hitchin, UK) was used based on the manufacturer’s instructions. A volume of 55.5 µL of sheared DNA was utilized for adapter ligation and USER excision followed by a size selection. Therefore, volumes of Ampure XP (Beckman Coulter GmbH, Krefeld, Germany) beads were adjusted for a 300 bp insert size. NEBNext^®^ Multiplex Oligos for Illumina^®^ (Index Primers Set 1) (New England Biolabs, Ltd., Hitchin, UK) were used for the PCR enrichment and sequencing was carried out on a NextSeq™ system (Illumina, San Diego, CA, USA).

### 2.15. Bioinformatic Analyses

Assembly of the sequences of G28 resulted in one contig that was arranged according to the genomic organization of phage T4, due to its circular permutation. The final contig was annotated using PROKKA 1.8 [40] followed by manual curation in Artemis [41]. The intergenic genome regions of the phage were analyzed with regard to putative transcriptional regulation elements using ARNOLD, based on [42]. A search for tRNA genes was done with the tRNAscan-SE program v1.2.1 [43] and ARAGORN v1.2.36 [44]. ResFinder 4.1 and VirulenceFinder 2.0 [45,46,47] were used to exclude the presence of genes coding for antibiotic resistance or virulence factors in the genome of G28.

To find differences to the wild type genome of E28, the sequence of E28.G28R3 was mapped using VarScan [48] and visualized with IGV [49].

The genome sequence of G28 was deposited at NCBI GenBank under the accession number MG867727. 

## 3. Results

### 3.1. Identification and Characterization of Bacteriophages Active against E. coli Field Isolates of Human and Animal Origin

#### 3.1.1. Six Phages were Isolated That Represent Different Morphological Subgroups of Myoviruses and Show Overlapping Host Ranges and Different Growth Characteristics 

**Figure 1:**
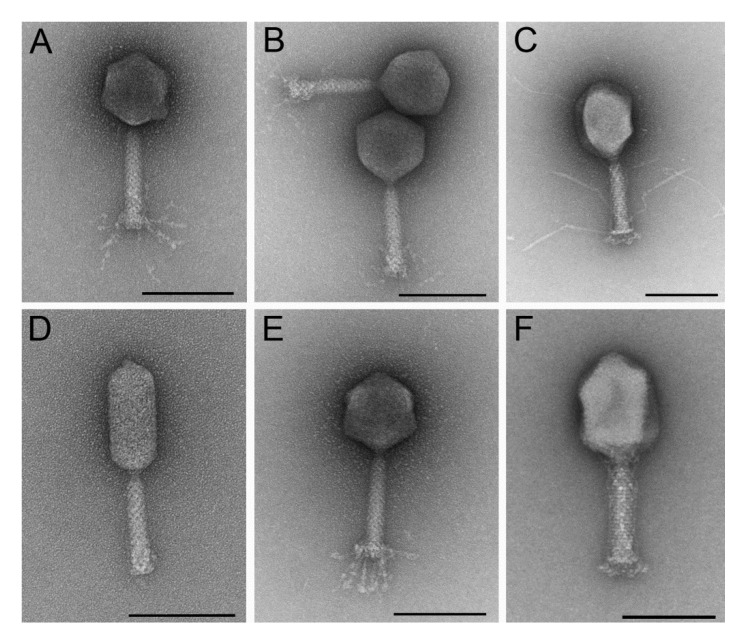
Morphology of phages. Transmission electron micrographs of negatively-stained phages EW2 (**A**), AB27 (**B**), TB49 (**C**), TriM (**D**), KRA2 (**E**), G28 (**F**) infecting *E. coli* strain E28 (Scale bars represent 100 nm).

**Figure 2:**
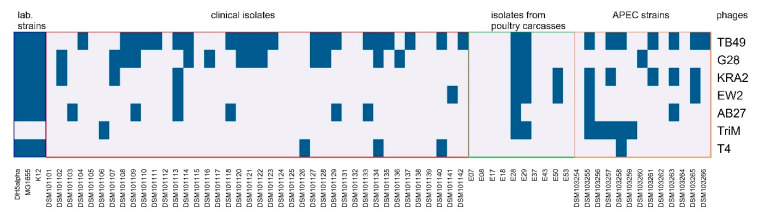
Host range analysis of phages. Serial dilutions of phage lysates were spotted on a lawn of the host bacteria. After overnight incubation plates were examined for plaques. Blue fields indicate the ability of phages to form plaques on the corresponding bacterial strains, gray fields represent no lysis. Experiments were performed at least twice.

For the composition of a phage cocktail, lytic phages were isolated from different environmental samples (Table S3). All phages revealed different morphological features of myoviruses (A1: EW2, AB27, KRA2; A2: G28, TB49; A3: TriM [50]) (Figure 1; Table S5). The phages form clear, small plaques (1–2 mm diameter) on a lawn of E28. Only the plaques of KRA2 are turbid on E28, but clear on isolation and production strains (Figure S1). The host range of the six phages was determined using 66 strains. Different serotypes, resistance patterns and virulence markers were identified in the strain collection as shown in Tables S1 and S2 indicating the diversity of the selected strains. As shown in Figure 2 and Table S6, TB49 showed the broadest host range and lysed 50% of the bacterial strains. The lytic activities of the other phages partially overlapped and ranged from 12 to 29%. Overall, the phages interacted with 67% of the tested strains. Neither a serotype specificity nor a connection to resistance pattern could be detected for phage sensitivity. None of the phages lysed only one specific serotype or all isolates of one serotype. However, it is noticeable that the phages KRA2 and TriM mainly lyse APEC strains. The lytic activity of the other phages is evenly distributed within the strain subgroups differing in their origin. 

One-step analysis revealed major differences with regard to their growth behavior (Figure S2, Table S7). EW2, TB49, KRA2, and G28 released progeny phages after 20–25 min, whereas reproduction of AB27 and TriM took 35–40 min. Average burst sizes ranged from 6 to 190 phage particles per cell. In tendency, an increased burst size coincided with a longer latent period. However, this was not true for phage AB27, which showed a low burst size and a long latent period. 

#### 3.1.2. All Bacteriophages Showed Lytic Activity under Conditions Relevant for In Vivo Investigation

We were further interested in investigating under which conditions other than the standard (37 °C, aerobic cultivation) the phages were able to form plaques. All phages were active at 42 °C and under microaerophilic conditions with similar activity compared to the standard conditions. In contrast, loss of activity could be observed at 10 °C and 20 °C. The activity of AB27, TB49, KRA2, and G28 at 20 °C was reduced less than 2 log units, whereas TriM and EW2 showed no activity at all. At an incubation temperature of 10 °C, only G28 remained active and was able to form plaques (Table S7).

We investigated the stability of the phages at different pH values, in order to estimate their potential survivability during the passage of the digestive system. As shown in Figure S3A all phages were stable in solutions with a pH range from 4 to 12 for 1 h. TriM, AB27, G28, and TB49 remained viable at pH 3 indicated by only minor reductions in titers under these conditions (Figure S3A). To evaluate the stability of phages during administration via drinking water, we analyzed the stability at room temperature for 24 h. Only slightly reduced titers could be observed (Figure S3B). The actual stability of phages diluted in tap water is presented in [21]. Furthermore, the phages EW2, TriM, AB27, TB49, and KRA2 were stable for at least six weeks at 6 °C in PBS or LB medium and therefore considered suitable for long-time storage without additives. The titer of G28 was reduced in PBS buffer at low NaCl concentrations, but remained stable in PBS buffer supplemented with 0.5% NaCl and in LB medium (Figure S3C).

### 3.2. Effectivity of the Phages against Model Strain E28

#### 3.2.1. *E. coli* Strain E28 was Shown to Allow Selective Quantification and Distinction from Other *E. coli* Strains, thus Being a Suitable Model Strain for In Vivo Experiments

A model strain for in vivo preventive phage application against APEC was selected by the following criteria: (a) ability to colonize in the chicken gut, (b) availability of different phages for the composition of an effective phage cocktail, (c) intestinal colonization resembling asymptomatic intestinal colonization of APEC strains before infection, and (d) selective quantification and discrimination between the target strain and other *E. coli* strains by markers such as metabolic or molecular characteristics. Since the occurrence of β lactamases in *E. coli* is frequently reported in livestock and thus represents a major public health problem, we analyzed 10 different ESBL-producing *E. coli* strains isolated from poultry meat for suitability. This strain selection varies in terms of serotypes and resistance pattern (Tables S1 and S2). However, the serotypes are intentionally not among those typically associated with colibacillosis [19] in order to ensure the model character of the strain. Analysis of the antibiotic resistance spectra identified *E. coli* strain E28 as a possible candidate that exclusively revealed resistance against kanamycin among this set of strains (Figure S4). Additionally, the evaluation of phenotype microarray data showed that E28 was amongst only two strains that were resistant to potassium tellurite (Figures S4 and S5). Therefore, the combination of kanamycin and potassium tellurite in a selective agar allowed the selective quantification and discrimination of this strain from other *E. coli* strains. A combination of different markers seems to be necessary based on the prevalence of single resistances [51]. E28 was thus further characterized as serotype O186:H34, carrying the β-lactamase gene *bla* CTX-M-14b, and the kanamycin resistance gene *aphA* (Tables S1 and S2, [39]). The respective genes provided targets for confirmation of the strain identity via PCR. All of the six phages formed clear, small plaques (1–2 mm diameter) on lawns of E28.

#### 3.2.2. Phage Combinations Show Dose Dependent Lysis of *E. coli* Model Strain E28 

**Figure 3:**
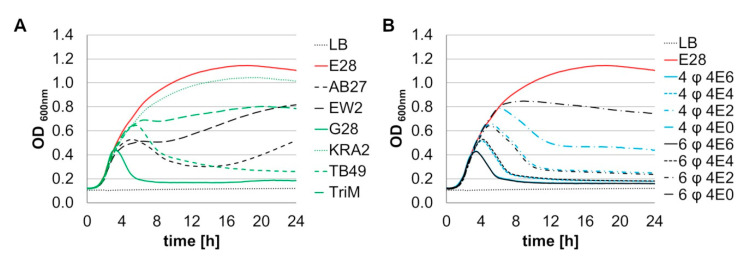
Efficacy of bacteriophages and phage combinations in inhibiting the growth of *E. coli* E28. Optical density of *E. coli* E28 cultures infected with (**A**) 4 × 10^6^ PFU of single phages AB27, EW2, G28, KRA2, TB49, and TriM and (**B**) 4 × 10^6^ PFU or hundredfold dilutions of phage combinations 4-phage (4 φ) (AB27, EW2, G28, an TB49) or 6-phage (6 φ) (4-phage preparation+ KRA2, TriM). Each experiment was performed twice with triplicate determinations. Mean values are presented without SD to maintain clarity; the average SD is given in Table S8.

An additional criterion for the selection of phages to be included in combined cocktails was their lytic activity against the target bacterium *E. coli* E28. Therefore, logarithmic phase growing E28 cultures were challenged with single phages approximately at MOI 0.1 or serial dilutions of phage combinations. G28 was most efficient in lysing a culture of E28. After 5 h, lysis was complete and no regrowth could be observed within 24 h. The phages AB27, EW2, TB49, and TriM showed inhibitory effects on bacterial growth, but no complete lysis of the culture. KRA2 presented the least ability to lyse a culture of model strain E28, indicated by a negligible reduction of bacterial growth (Figure 3A). The ability of the two phage preparations four-phage (EW2, TB49, G28 and AB27) and six-phage (EW2, TB49, G28, AB27, KRA2, and TriM) to lyse E28 cultures was determined at different MOIs (0.1 ≙ 4E6, 0.001 ≙ 4E4, 0.00001 ≙ 4E2, 0.0000001 ≙ 4E0) (Figure 3B). To clarify whether a higher number of phages can provide an advantage through higher diversity or whether a high number of phages is rather disadvantageous because the most effective phages are diluted, the two least effective phages (TriM and KRA2) were removed in the four-phage preparation. The highest MOI showed the maximum lytic effect on E28 cultures for both the four- and six-phage preparation. The ability to lyse the bacterial culture was found to be dose dependent. At MOIs of 0.1 to 0.00001 no differences in the lytic ability of the two preparations could be observed with one exception. Only at MOI 0.00001 the four-phage preparation was slightly more effective than the six-phage preparation. In a subsequent in vivo study, one animal experiment was conducted with each of the two cocktails [21], confirming these findings.

#### 3.2.3. The Six-Phage Preparation Effectively Reduces Biofilm Formation by E28 and DH5α but Not by ECOR10

**Figure 4:**
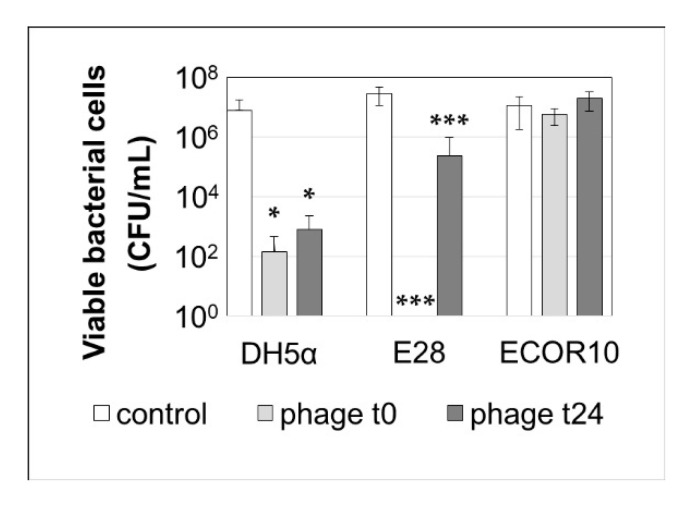
Effect of the 6-phage preparation on the formation of *E. coli* biofilms. Bacterial cultures were infected with 1 × 10^6^ PFU/mL of the phage preparation at the beginning of the experiment (t0) and after 24 h (t24). Bacterial counts were determined after 48 h. Data are presented as mean with SEM of triplicate determinations (*n* = 4). Statistical analysis was performed using two-way ANOVA and Bonferroni corrected post-hoc tests for detection of significant differences. * *p* < 0.05 and *** *p* < 0.001.

To study the effect of the six-phage (EW2, TB49, G28, AB27, KRA2, and TriM) preparation on the establishment and maintenance of *E. coli* biofilms, three bacterial strains were investigated. DH5α and E28 were sensitive to the phage preparation, while *E. coli* ECOR10, a biofilm forming strain from a previous study [52], was insensitive to all phages in the preparation. The results shown in Figure 4 indicated that the six-phage preparation completely prevented the formation of a biofilm by E28, when applied at the start of the experiment, and significantly reduced the amount of biofilm formed by DH5α but had no effect on the biofilm formation by the non-sensitive host ECOR10. When the phage preparation was added to an established biofilm, further proliferation of the biofilm was prevented for both sensitive strains DH5α and E28. As above, no effect was observed on the insensitive host ECOR10 and the phages were not able to disperse the already existing biofilm of all three tested strains.

### 3.3. Genomic Analysis of G28

Since G28 has been shown to be the most efficient phage to control E28 and the only one isolated on this strain, we were particularly interested in the genetic properties of this phage, since it may also have the greatest impact in in vivo experiments.

Sequencing of the circularly permuted genome of phage G28 resulted in a final genome size of 170,121 bp. Further analysis revealed characteristic gene clusters for head and tail structure, replication, and host cell lysis. Generally, strong similarities to phage PE37 at the nucleotide level and in regard to its genomic organization were detected by genome comparison visualized using Easyfig [53,54]. Both phages taxonomically belong to the *Tequatrovirus* and show typical features of that group. Compared to T4, one major difference was detected in the gene cluster for structural components (Figure S6, marked in green). Two gene products (locustags G28_00253 and G28_00254) with conserved domains for a putative peptidase (pfam13884) and a putative phage tail fiber adhesion protein (pfam05268), respectively, revealed no or only weak similarities to their homologs in the genome of T4. As described by Pirnay et al. we examined the phage genome in regard to specific requirements for safe application [55], which did not reveal any hints at lysogeny and therefore G28 was considered to be lytic. Furthermore, G28 did not encode any toxins, virulence factors or antibiotic resistance-mediating genes (Supplementary Materials Files S1 and S2).

### 3.4. Phenotypic and Genotypic Characterization of Phage-Resistant Variants of E. coli E28

#### 3.4.1. Some Phage-Resistant Strain E28 Variants Show Decreased Growth, Reduced Metabolic Ability, and No Motility

After infection of E28 with single phages of the cocktail at MOI 100, emerging phage-resistant variants were isolated and further analyzed for their susceptibility to other phages of the cocktail to gain more insight into phage-host interactions. We observed equal resistance patterns for resistant isolates that originated from treatment with one phage. Results of one representative phage resistant mutant are shown in Table 1. Phage-resistant variants against KRA2/EW2 and G28/TB49 showed almost similar resistance patterns but no cross resistance. E28.TriMR3 was additionally resistant or less sensitive to G28 and TB49. E28.AB27R2 had only a reduced sensitivity to AB27 and stayed sensitive to all other phages. Results of one representative phage-resistant variant are shown in Table 1. Overall, all phage-resistant variants investigated stayed susceptible to at least one phage of the cocktail (Table 1). 

Furthermore, we analyzed different mutants with regard to their growth behavior. We showed that the mutants E28.G28R3 and E28.TB49R2c had shown decreased growth compared to the wild type (Table 1). To determine whether changes in the carbon consumption were responsible for this finding, we analyzed the oxidation rates of 95 different carbon sources using the OmniLog technology. The phage-resistant variants E28.AB27R2, E28.KRA2R3, E28.EW2R3, and E28.TriMR3 displayed very similar metabolic fingerprints compared to E28 WT. With one exception (E28.EW2R3: α-D-Lactose) the mutants use the same carbon sources as E28 WT. In contrast, the phage-resistant variants E28.G28R3 and E28.TB49R2c showed reduced metabolic activity in 28 and 25 cases, respectively, but oxidized eight carbon sources to a higher extent than the wild type (Table 1 and Table S9). Additionally, we observed changes in the growth behavior on agar plates in regard to swimming motility of some mutants. Most mutants resistant against the phages G28 and TB49, including E28.G28R3 and E28.TB49R2c, showed no motility at all (Table 1). Those results were verified by light microscopy, where mutant cells also revealed a highly reduced activity, mobility, and cell size compared to the wild type. In contrast, we detected an increased swimming motility in the mutant resistant against TriM. 

#### 3.4.2. E28.G28R3 Shows Altered Adsorption Behavior and Changes in the Genome

**Figure 5:**
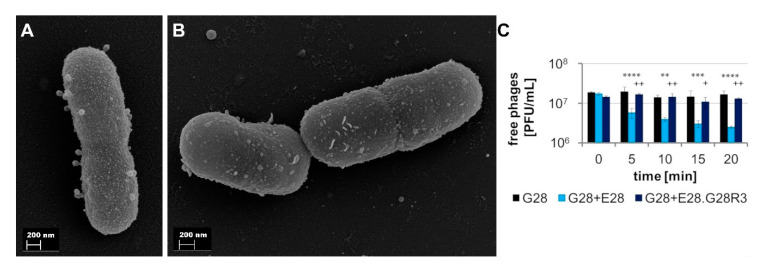
Comparison of adsorption of G28 to wild type E28 and phage-resistant mutant E28.G28R3. FESEM of E28 wild type (**A**) and mutant E28.G28R3 (**B**) 20 min after addition of phage G28. (**C**) Adsorption of G28 on E28 wild type and mutant E28.G28R3. Mean and SD of three experiments with duplicate determinations are shown. Statistical analysis was performed using two-way ANOVA and bonferroni corrected post-hoc tests for detection of significant differences. Stars indicate comparison of “G28” and “G28 + E28”, crosses signify the comparison of “G28 + E28” and “G28 + E28.G28R3” (^+^ *p* < 0.05, ^++/^** *p* < 0.01, *** *p* < 0.001 and **** *p* < 0.0001).

Mutant E28.G28R3 was selected for further analyses as G28 was considered to be most relevant for subsequent in vivo experiments based on the high lytic efficacy of this phage against E28. To get more insights into the resistance mechanism of E28.G28R3, we studied the ability of G28 to adsorb to E28.G28R3 cells. While the numbers of free phages added to the wild type culture decreased continually and reached the minimum after 20 min, phages were unable to adsorb to the mutant E28.G28R3 as indicated by the constant number of free phage particles (Figure 5C). Adsorption resistance could be confirmed by FESEM (Figure 5A,B).

**Figure 6:**
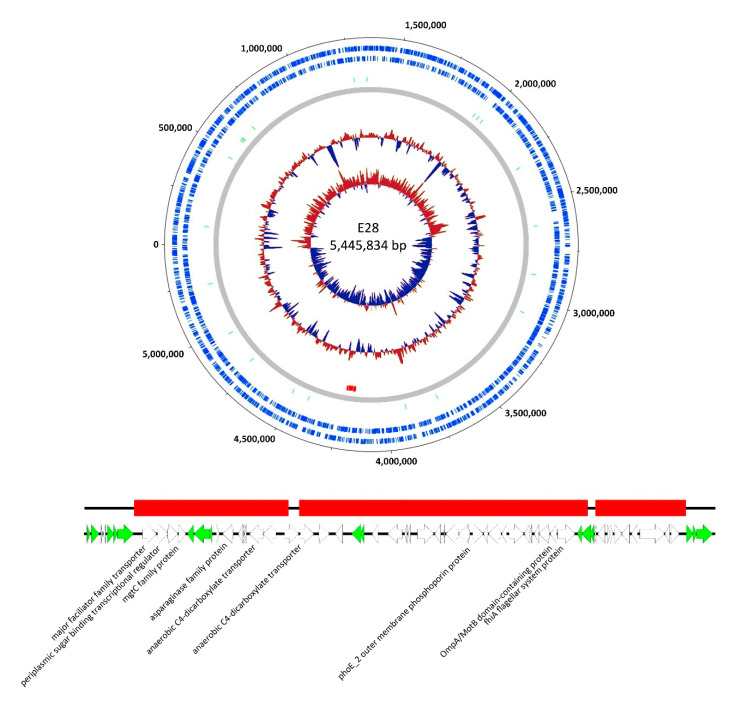
Deleted region in the genome of phage-resistant *E. coli* E28.G28R3 harboring genes putatively necessary for phage adsorption. The location of the deleted region is marked in red in the genome of wild type E28. Genes marked in green are linked to putative transposase activity and regulation. A detailed map of the deletion was visualized with EasyFig.

The data on the adsorption behavior of phage G28 indicated a putative change in the phage receptor. In order to get insights into those genomic changes causing phage resistance against G28 and a hint on the putative receptor itself, the whole genome of E28.G28R3 was sequenced using Illumina technologies and compared to the wild type genome of E28. Detailed analysis revealed a deletion of about 55 kb in the genome of E28.G28R3, located between genomic position 4,175,477 bp and 4,230,077 bp with short interruptions (Figure 6). This region is flanked by genes encoding putative transposases or repressor-like proteins, some transposase-like genes were also detected in the missing region itself. Altogether, those findings indicate that this deletion might be based on transposase activity. Among others, this region also contained genes coding for different transporter proteins, a conserved FlhA-like protein (COG1298), a MotB domain protein (COG1360) belonging to the OmpA-family and a putative outer membrane protein (COG3203) of the OmpC family. We hypothesize that the absence of one of these genes might be responsible for the mutated phenotype described.

## 4. Discussion

Replacing or supplementing antibiotics by phage applications has high potential for the prevention of losses in animal farming to control the spread of MDR via the food chain [14,56,57].

Although some experimental studies on the treatment of *E. coli* infections in poultry by phage application could successfully prevent or reduce mortality of artificially infected animals, none of these experimental setups were appropriate for prevention of disease and economic losses under commercial farming conditions [58]. Therefore, preventive application via the drinking water might be feasible under commercial conditions, rather than a curative approach. It might reduce the gut carriage of avian pathogenic *E. coli*, keeping them below the minimum infective dose in the inhaled air and thus preventing clinical outcomes that require antibiotic treatment. 

### 4.1. Selection of an E. coli Model Strain and Cocktail Composition for a Subsequent Animal Experiment Testing Phage Application against Intestinal Colonization of APEC

To test the efficacy of the prevention or reduction of intestinal colonization of extraintestinal pathogenic *E. coli* variants we used a chicken model of intestinal colonization with an apathogenic traceable model strain of *E. coli* where phages were applied in a continuous manner via the drinking water as presented by Kittler et al. [21]. In this follow up study the impact of the four-phage and six-phage cocktail in vivo is described. *E. coli* E28 was identified as a suitable model bacterium. Traceability could be ensured by a combination of different markers and the strain colonized the gut of chickens asymptomatically [21]. Six phages were identified that proved to be the most effective against E28. Two cocktails were composed from the single phages. The first cocktail consisted of six different phages, which reflected the greatest possible diversity; the second cocktail was limited to the four most effective phages, based on their ability to suppress growth of E28. The composition of the cocktails was based on (a) diversity of the phages, (b) lytic efficiency, and (c) the production of these phages has to be easily achieved on well-characterized hosts.

### 4.2. Cocktail Composition Based on Diversity of Phages

An important criterion for a phage composition is the host range of the individual phages [59] ideally with partially overlapping and supplementary host ranges. No relationship between phage susceptibility and serotype or antibiotic resistance phenotype was found in the examined host strain collection. The specificity of the cocktail was focused on model strain E28, which was used to evaluate the preventive use of the phage cocktail for reduction of asymptomatic colonization of extraintestinal pathogenic *E. coli*. The host ranges of the phages were determined on a diverse *E. coli* strain collection including clinical ESBL-producing *E. coli*, ESBL-producing *E. coli* isolated from poultry carcasses and APEC strains. As shown by others, phages show similar lytic activity on ESBL-producing bacteria and bacteria without ESBL production [60,61,62]. The morphologically T4-like phages TB49 and G28 exhibited broad spectra of lytic activity, whereas the other phages of the cocktail had relatively narrow spectra that targeted E28 very specifically. Although all phages belong to the group of myoviruses, they exhibit diversity in terms of their host specificity. In contrast to other studies, we could not find any serotype specificity of our phages. Oliveira et al. composed a phage cocktail with coliphages effectively lysing primarily APEC strains with the most prevalent serotypes O2, O5, O78, and O88 [19]. However, KRA2 and TriM show a prevalence to lyse APEC strains, whereas they hardly lyse clinical isolates, making them attractive for a commercial phage cocktail to prevent infections with pathogenic *E. coli* in flocks and were therefore included in the six-phage cocktail. 

Within the *Caudovirales* there is a remarkable diversity of growth characteristics, which is among others reflected in a burst size of 30–200 PFU/infected cell [63,64,65,66,67,68]. Except TB49 and AB27, which produce 6–7 phage particles per infected cell, all determined burst sizes range within this scale. Until now, no optimal latent periods and burst sizes have been identified for phages in therapeutic and preventative applications, but phages showing fast replication and high burst sizes are generally preferred [69]. A fast replication can compensate a smaller burst size, which, in this study was true for G28. Variations in environmental factors like temperature, pH, medium, cation availability, host, host density, and the physiological status of the host all influence the latent period and burst size of different phages, and thus make these characteristics hard to compare with in vivo situations [70,71]. By combining phages with different growth characteristics, we hoped to increase the chance of providing efficient in vivo phages, since no in vitro model is yet available that allows reliable predictions.

### 4.3. Phages Show Sufficient In Vitro Stability for Testing In Vivo

Phages for therapeutic or prophylactic purposes should be highly stable and remain viable in a wide range of potential environments. The characterized phages met the requirements with regard to stability aspects for storage and application in drinking water. As known from the literature, phage stability at low pH is limited [72,73]. During stomach passage, phages are exposed to an acidic environment for about 1–1.5 h in chickens and a pH of about 2.6 [74]. A negative influence on phage stability by bile salts is unlikely since bile salts at concentrations ranging from 4 to 10 mM were shown to have a minor impact on phage stability [75]. The stability of the phages of this study seemed to be sufficient to reach the *E. coli* model strain E28 in the chicken intestinal tract in subsequent in vivo trials [76,77]. 

### 4.4. In Vitro Determination of an Efficient Cocktail against E28

Lysis curves provide information on the antimicrobial activity of phages in liquid environments, which can be different from the activity against cells in overlay agar due to a different expression of surface receptors or an effect of host density around the phages. We found major differences between lytic activity in overlay agar and in liquid medium (Figure S1 and Figure 3). Phages produced clear plaques on a lawn of E28, but incomplete lysis was observed at MOI 0.1, especially for KRA2. These findings can hardly be explained by differences in the growth characteristics of phages. Both the phage which displayed the highest bactericidal activity (G28) and the phage with negligible bactericidal activity against E28 (KRA2) in broth culture, showed comparable burst sizes and reproduction times. Even the high burst size of around 200 progeny phages per cycle of infection, as determined for TriM, did not lead to complete lysis [78]. Kim et al. found that incomplete lysis in liquid culture and turbid plaques could be explained by a proportion of the initial host population being insensitive to the phage [79]. Another study found an indication of physiological and genetic modification of the host cell during phage infection, which resulted in transient phage resistance and incomplete lysis [80]. Unlike some of the single phages, our in vitro results demonstrate that the phage cocktail was highly efficient in lysing host bacteria in broth culture. In contrast to other studies dealing with the efficacy of phage cocktails [81], no synergistic effect of the phages could be detected, due to the high efficiency and absence of regrowth in the presence of G28. It was shown by Henry et al. that a phage isolated on the target bacterium was more effective in lysing this strain than phages from a phage collection [82], which was confirmed in our study. Due to economical and practical conditions of broiler production, the efficiency of phages was tested at MOIs below one to reflect restricted phage concentration during practical application due to mixing of individual phages and the dilution in drinking water. Accordingly, phage concentrations in field trials will not exceed 10^7^ PFU/mL. For reduction of *E. coli* that occurs in high concentrations in the chicken gut (10^6^–10^8^ CFU/g feces), a cocktail would have to be highly efficient even at low MOIs [83], as presented in our in vitro studies. 

In addition, we showed that the six-phage preparation can efficiently prevent the formation of a biofilm produced by E28 in vitro and significantly reduce an existing biofilm as reported by others [84,85,86,87]. Biofilms are a problem especially in the pipe systems of drinking troughs, as they are difficult to eliminate by disinfectants or cleaning [88]. A spreading of biofilm forming *E. coli* could be prevented by application of phages via drinking water. Phages can kill planktonic bacteria to prevent or slow down the establishment of a biofilm [89,90]. Some phages encode polysaccharide depolymerases that can attack the intercellular polysaccharide matrix of biofilms and thus are able to combat already established biofilms [91,92]. Our experiments showed that the six-phage preparation was able to prevent the establishment of a biofilm of E28 but was not able to completely disperse already existing biofilms. Consequently, phage preparations with this property should be used as a preventive rather than as an intervening measure.

Our follow-up study also showed that a high in vitro efficiency is not necessarily able to simulate the complex interplay of intestinal colonization in chickens, which agrees with findings of previously published results [18,93]. An explanation for the different in vitro findings presented here and in vivo results in the corresponding animal experiments [21] could be the replication of the phages in other *E. coli* strains besides model strain E28. The possibility that the phages were not stable or could not replicate under in vivo conditions seems unlikely based on the determined amount of phage in the feces. However, it is possible that the amount of phage applied did not lead to optimal conditions for the reduction of the model strain E28. 

### 4.5. Phages Were Composed to Prevent the Development of Resistance

To prevent the emergence of phage resistant bacterial populations, application of a phage cocktail composed of phages using different host receptors is generally preferred compared to the use of single phages [10,94,95,96]. Our results demonstrate that growth of phage-resistant variants was prevented in in vitro experiments. We composed the cocktail of phages belonging to different morphotypes (A1–A3 morphology), showing different growth behavior, host ranges, and lytic efficacy to increase the probability of different receptors being used and efficiency in vivo. Our results suggest that the six characterized phages might use different receptors for host adsorption, as shown in Table 1. Variants resistant to one phage were still sensitive to at least another phage in the cocktail. 

Coliphages use a broad range of host receptors including O antigen of LPS, outer membrane proteins, pili, fimbriae, and flagella antigens [97]. Trojet et al. identified host receptors for a number of phages belonging to the subfamily Tevenvirinae. T4 uses OmpC and LPS as receptors when adsorbing to *E. coli* K-12 [28,98,99,100]. In contrast to G28 and TB49, T4 is not able to infect E28. We thus hypothesize differences in the receptor specificity compared to T4 (Figure 1). To understand the action of resistance of E28 against G28, genetic mapping of the spontaneous mutant was performed. Among other genes, mutant E28.G28R3 lacks the genes coding for a MotB domain protein belonging to the OmpA family and a putative outer membrane protein of the OmpC family, therefore it can be hypothesized that one of these proteins might be necessary for adsorption of G28. It is likely that the phenotype of reduced swimming motility and reduced growth is a consequence of the deletion of the receptor region, but further studies are necessary to clarify this issue. However, while a direct association of this phenotype with phage resistance seems unlikely, reduced motility and carbon usage might result in decreased colonization ability of the mutants E28.G28R3 and E28.TB49R2c (Table 1) [101,102,103,104,105,106,107]. The results of the following in vivo study underline the effect of combined phage application on the prevention of resistance [21].

## 5. Conclusions

In the present study we characterized six phages suitable for preventing infections with pathogenic and/or multidrug resistant ESBL-producing *E. coli*. We demonstrated that the phages showed lytic activity against a broad range of ESBL-producing and avian pathogenic *E. coli* isolates. The morphological diversity and the bactericidal activity against the model *E. coli* strain E28 indicated that the characterized phages are promising candidates for subsequent in vivo efficacy studies. Analysis of phage resistant mutants indicated advantages of a combined application of four- or six-phages. 

## Figures and Tables

**Table 1:** Summarized comparison of sensitivity towards other phages, metabolic activities, swimming motilities, and growth of phage-resistant variants vs. the E28 wild type strain.

	Strain	E28 (WT)	E28.AB27R2	E28.EW2R3	E28.G28R3	E28.KRA2R3	E28.TB49R2c	E28.TriMR3
**sensitivity against phages**								
G28		+	+	+	−	+	(−)	+
EW2		+	+	−	(−)	−	(−)	+
KRA2		+	+	+	+	−	+	+
AB27		+	(−)	+	+	+	+	+
TriM		+	+	+	−	+	(−)	−
TB49		+	+	+	(−)	+	−	+
**metabolic activity**								
negative		36	38	36	48	36	48	36
positive		51	47	50	6	51	3	51
reduced			2	0	25	0	28	0
increased			0	1	8	0	8	0
**growth behavior**								
swimming motility diameter [mm]		26.3 ± 4.5	24.0 ± 7.7	19.0 ± 9.0	1.0 ± 0.0	20.0 ± 5.4	1.0 ± 0.0	33.3 ± 4.9
growth (OD_600nm_, 24 h at 37 °C)		1.33 ± 0.04	1.03 ± 0.01	1.33 ± 0.05	0.53 ± 0.02	1.34 ± 0.05	0.59 ± 0.03	1.34 ± 0.06

Serial dilutions of phage lysates were spotted on double layer agar plates containing the phage-resistant variants and examined for plaque formation (+ = sensitive, − = insensitive, (−) = reduced sensitivity (EOP < 10^−4^)). Metabolic activities of phage-resistant variants were compared with that of *E. coli* E28 wild type (WT) for different carbon sources and rated as follows: negative = no metabolic activity, positive = metabolic activity (comparable to that of the E28 WT), reduced = lower metabolic activity than E28 wild type, increased = higher metabolic activity than E28 wild type. Two independent experiments were performed. For swimming motility, the diameter of colonies derived from culture on 0.3% agar plates was measured after incubation at 37 °C for 18 h. Mean and SD of three independent experiments were calculated. A course of optical density was recorded for 24 h at 37 °C to compare growth of phage-resistant mutant strains with E28 WT. The mean and SD of triplicate determinations is shown.

## Supplementary Materials

The following are available online at https://www.mdpi.com/1999-4915/12/12/1470/s1, Table S1: *E. coli* strains used for phage isolation, characterization and propagation. Table S2: Serogenotypes, resistance und virulence markers of *E. coli* strains, analyzed by PanType. Table S3: Phages used in this study. Table S4: Contents of antibiotic discs used for susceptibility testing. Table S5: Mean dimensions of phage particles with SD., Figure S1: Plaque morphology of coliphages on E28. Table S6: The percentage amount of strains lysed by each phage. Figure S2: Growth of coliphages. Table S7: Growth of coliphages. Figure S3: Stability of phages stored under different conditions. Figure S4: Antibiotic resistance patterns and resistance against potassium tellurite of 10 ESBL-producing *E. coli* isolates. Figure S5: Results of the phenotypic array using Gen III Microplates. Table S8: Average SD corresponding to Figure 3, Figure S6: Genome structure of phage G28 in comparison to the related phages PE37 and T4. Table S9: Comparison of metabolic activity of phage-resistant variants and the wild type strain, Supplemental Methods (“Phage isolation, purification and propagation”, “Morphology of phages and analysis of phage bacteria interactions”, “One-step growth assays”, “In vitro biofilm model”, “DNA isolation from phages”), File S1 “Virulencefinder Results”, File S2 “ResFinder phenotype results”.
