# Supplementary material for: In Vitro Evaluation of a Phage Cocktail Controlling Infections with Escherichia coli
Source: Viruses. 2020 Dec 19;12(12):1470. doi: 10.3390/v12121470 (PMC7768485; doi:10.3390/v12121470)
Supplement: Supplementary file 1 [file viruses-12-01470-s001.zip › viruses-974160-1/Supplements/viruses-974160-supplementary18122020.pdf]

viruses MDPI

Supplementary Materials

Table S1: *E. coli* strains used for phage isolation, characterization and propagation (^{NM} = non motile, ^{NT} = non-typeable, ND = no data, risk group = RG, + = positiv, - = negative)

strain designation	origin	date of isolation	geographical origin	serotype	remarks
DSM 498 (K12)	ND	ND	ND	O16:H48	laboratory strain, RG 1
DSM 613 (B)	ND	ND	ND	O7:H^{NT}	laboratory strain, RG 1
DSM 6897 (DH5α)	ND	ND	ND	ND	laboratory strain, $\lambda^{(-)}$, RG 1
DSM 18039 (MG1655)	ND	ND	ND	ND	laboratory stran, F$^{(-)}$, $\lambda^{(-)}$, RG 1
DSM 101101	human urine	25-09-2016	Germany	O25:H4	ESBL, RG 2
DSM 101102	human	25-09-2016	Germany	O102:H^{NT}	ESBL, RG 2
DSM 101103	human	25-09-2016	Germany	O^{NT}:H4	ESBL, RG 2
DSM 101104	human urine	29-09-2015	Germany	O7:H5	ESBL, RG 2
DSM 101105	human	29-09-2015	Germany	O^{NT}:H4	ESBL, RG 2
DSM 101106	human urine	28-09-2015	Germany	O75:H9 var. 7	ESBL, RG 2
DSM 101107	human urine	28-09-2015	Germany	O^{rough}:H6	ESBL, RG 2
DSM 101108	human urine	30-09-2015	Germany	O25:H4	ESBL, RG 2
DSM 101109	human urine	30-09-2015	Germany	O25:H4	ESBL, RG 2
DSM 101110	human	01-10-2015	Germany	O^{NT}:H9 var. 7	ESBL, RG 2
DSM 101111	human	01-10-2015	Germany	O25:H4	ESBL, RG 2
DSM 101112	human	01-10-2015	Germany	O25:H4	ESBL, RG 2
DSM 101113	human	18-09-2015	Germany	O25:H4	ESBL, RG 2
DSM 101114	human	22-09-2015	Germany	O25:H4	ESBL, RG 2
DSM 101115	human	23-09-2015	Germany	O75:H9 var. 7	ESBL, RG 2
DSM 101116	human	24-09-2015	Germany	O^{NT}:H15	ESBL, RG 2
DSM 101117	human urine	11-09-2015	Germany	O25:H4	ESBL, RG 2
DSM 101118	human	12-09-2015	Germany	O25:H4	ESBL, RG 2
DSM 101120	human	17-09-2015	Germany	O25:H4	ESBL, RG 2
DSM 101121	human	09-09-2015	Germany	O^{NT}:H1	ESBL, RG 2
DSM 101122	human	10-09-2015	Germany	O7:H5	ESBL, RG 2
DSM 101123	human	10-09-2015	Germany	O7:H5	ESBL, RG 2
DSM 101124	human urine	29-08-2015	Germany	O^{NT}:H15	ESBL, RG 2
DSM 101125	human	28-08-2015	Germany	O^{NT}:H9 var. 7	ESBL, RG 2
DSM 101126	human urine	29-08-2015	Germany	O75:H9 var. 7	ESBL, RG 2
DSM 101127	human	02-09-2015	Germany	O25:H4	ESBL, RG 2
DSM 101128	human	02-09-2015	Germany	O25:H4	ESBL, RG 2
DSM 101129	human urine	03-09-2015	Germany	O25:H4	ESBL, RG 2
DSM 101131	human urine	22-08-2015	Germany	O25:H4	ESBL, RG 2
DSM 101132	human	22-08-2015	Germany	O^{rough}:H4	ESBL, RG 2
DSM 101133	human	22-08-2015	Germany	O^{NT}:H18	ESBL, RG 2
DSM 101134	human urine	23-08-2015	Germany	O7:H5	ESBL, RG 2
DSM 101135	human urine	23-08-2015	Germany	O25:H4	ESBL, RG 2
DSM 101136	human urine	23-08-2015	Germany	O25:H4	ESBL, RG 2
DSM 101137	human	23-08-2015	Germany	O^{NT}:H18	ESBL, RG 2
DSM 101138	human	27-08-2015	Germany	O1:H6	ESBL, RG 2
DSM 101139	human	24-08-2015	Germany	O1:H6	ESBL, RG 2

DSM 101140	human	24-08-2015	Germany	O^{NT}:H6	ESBL, RG 2
DSM 101141	human	23-08-2015	Germany	O15:H1	ESBL, RG 2
DSM 101142	human	27-08-2015	Germany	O^{NT}:H4	ESBL, RG 2
DSM 103242 (E07)	chicken carcass	17-09-2012	Netherlands	O138:H48	ESBL, RG 2
DSM 103243 (E08)	chicken carcass	17-09-2012	Netherlands	O25:H48	ESBL, RG 2
DSM 103244 (E17)	chicken carcass	17-09-2012	Belgium	O38:H39	ESBL, RG 2
DSM 103245 (E18)	chicken carcass	17-09-2012	Belgium	O38:H39	ESBL, RG 2
DSM 103246 (E28)	chicken carcass	08-10-2012	Germany	O186:H34	ESBL, RG 2
DSM 103247 (E29)	chicken carcass	08-10-2012	Germany	O88·H7	ESBL, RG 2
DSM 103248 (E37)	chicken carcass	08-10-2012	France	O162:H10	ESBL, RG 2
DSM 103249 (E43)	chicken carcass	08-10-2012	France	O^{NT}:H10	ESBL, RG 2
DSM 103250 (E50)	chicken carcass	13-01-2014	Netherlands	O^{NT}:H25	ESBL, RG 2
DSM 103251 (E53)	chicken carcass	13-01-2014	Netherlands	O91:H7	ESBL, RG 2
DSM 103254	chicken air sac	12-07-1999	Germany	O78	APEC; astA -, CDTIII -, CNF1/2 -, FyuA -, irp2 -, hlyA -, aer +, tsh +, fimC +, papC +, hlyE -, stx2f -
DSM 103255	chicken peritoneum	12-10-1999	Germany	O78	APEC; astA +, CDTIII -, CNF1/2 -, FyuA +, irp2 +, hlyA -, aer +, tsh +, fimC +, papC -, hlyE -, stx2f -
DSM 103256	chicken	11- 1999	Germany	O2:K1	APEC
DSM 103257	chicken wattle	11-1999	Germany	O2:K1	APEC
DSM 103258	chicken	24-02-2000	Germany	O2:K1	APEC
DSM 103259	chicken bone marrow	24-02-2000	Germany	O2	APEC
DSM 103260	chicken heart blood	10-04-2000	Germany	O1:H^{NM}	APEC; astA +, CDTIII -, CNF1/2 -, FyuA +, irp2 +, hlyA -, aer +, tsh +, fimC +, papC +, hlyE -, stx2f -
DSM 103261	chicken	05-07-2001	Germany	O2:K1	APEC
DSM 103262	chicken	05-07-2001	Germany	O1:K1	APEC
DSM 103263	chicken heart blood	10-07-2001	Germany	O78:K80	APEC; aerA+, tsh+
DSM 103264	chicken	29-05-2001	Germany	O1:K1	APEC
DSM 103265	dove	12-01-2004	Germany	O1:H15	APEC; Col-, Hly-,
DSM 103266	chicken	01-03-2005	Germany	O2	APEC; astA-, iss +, irp2 +, papC -, iuc D +, tsh +, vat -, cvi/cva +
E64	pig farm	2014	Germany	ND	ND
ECOR10	human	ND	USA	O6:H10	group A strain, RG 1
ECOR13	human	ND	Sweden	O173:H^{NM};	group A strain, RG 1
ECOR17	pig feces	ND	Indonesia	O106:H^{NM}	group A strain; RG 1
ECOR28	human feces	ND	USA	O104:H^{NM}	group B1 strain; RG 1
ECOR47	sheep	ND	New Guinea	O^{NT}:H18	group D strain, RG 2

Table S2: Serogenotypes, resistance und virulence markers of *E. coli* strains, analyzed by PanType

	**strain**	**E07**	**E08**	**E17**	**E18**	**E28**	**E29**	**E37**	**E43**	**E50**	**E53**
**Serogenotype**	**O-Serotype**										O91
	**H-Serotype**	fliC H48	fliC H48	fliC H39	fliC H39	fliC H34	fliC H7	fliC H10	fliC H10	fliC H25	fliC H7
**Resistance associated genes against**	**Aminoglycoside -antibiotics**	aadA1, aadA2	aadA1, aadA2	strB	strA, strB	aadA2, aphA, strA, strB		strB	aadA1, aadA2		
	**β-lactam-antibiotics**	blaSHV	blaSHV	blaTEM	blaTEM	blaCTX-M9	blaTEM	blaTEM	blaTEM, blaCTX-M9	blaCTX-M1, blaCTX-M15	blaCTX-M1, blaCTX-M15
	**Chlor-amphenicol**	cmlA1	cmlA1						cmlA1		
	**Macrolid-antibiotics**										
	**Tetracyclines**	tetA	tetA	tetA	tetA	tetB			tetB		
	**Sulfonamide**	sul3	sul3			sul1, sul2		sul2	sul3	sul2	sul2
	**Trimethoprim**							dfrA14	dfrA1		
**genes encoding virulence factors**	**Adhesins**										
	**Fimbriae**					prfB					lpfA
	**secretion system**										
	**Autotransporter**										
	**Toxins**	cma	cma	astA	astA	astA	astA		cma, mchF	cma	cma
	**miscellaneous**	hemL, intI1, iroN, iss	hemL, intI1, iroN, iss	hemL, iss	hemL, iss	hemL, ireA	hemL, iss	hemL	hemL, intI1, intI2, iroN, iss	hemL, ireA, iroN, iss	hemL, iroN, iss

	**strain**	**DSM 101102**	**DSM 101104**	**DSM 101105**	**DSM 101111**	**DSM 101112**	**DSM 101113**	**DSM 101114**	**DSM 101120**	**DSM 101121**	**DSM 101122**
**Serogenotype**	**O-Serotype**										
	**H-Serotype**	fliC H6	fliC H5	fliC H4	fliC H4	fliC H4	fliC H4	fliC H4	fliC H4	fliC H1, fliC H46	fliC H5
**Resistance associated genes against**	**Aminoglycoside -antibiotics**	aadA4, strA, strB	strB	aadA1, aphA, aadA4	aac6, aac6Ib, aadA4	aac6, aac6Ib, aadA4, strA, strB	aac6, aac6Ib, aadA4	strA, strB, aadA4	aadA4	strB	strB
	**β-lactam-antibiotics**	blaCTX-M1, blaCTX-M15	blaCTX-M1, blaCTX-M15, blaTEM	blaCTX-M1, blaCTX-M15, blaOXA-1	blaCTX-M1, blaCTX-M15, blaOXA-1	blaCTX-M1, blaCTX-M15, blaOXA-1	blaCTX-M1, blaCTX-M15, blaOXA-1, blaTEM	blaCTX-M1, blaCTX-M15, blaTEM	blaCTX-M1, blaCTX-M15, blaTEM	blaCTX-M1, blaCTX-M15, blaTEM	blaCTX-M1, blaCTX-M15, blaTEM
	**Chlor-amphenicol**	floR		catA1	catB3	catB3	catB3				
	**Macrolid-antibiotics**	mphA, mrx	mphA, mrx		mphA, mrx	mphA, mrx	mphA, mrx	mphA, mrx	mphA, mrx	mphA	
	**Tetracyclines**	tetA, tetB		tetB	tetA	tetA		tetA	tetA, tetB	tetB	
	**Sulfonamide**	sul1, sul2	sul2	sul1, sul2	sul1	sul1, sul2	sul1	sul1, sul2	sul1	sul2	sul1, sul2
	**Trimethoprim**	dfrA17, dfrA19		dfrA7, dfrA17, dfrA19	dfrA17, dfrA19	dfrA17, dfrA19	dfrA17, dfrA19	dfrA5, dfrA17, dfrA19	dfrA17		
**genes encoding virulence factors**	**Adhesins**		iha		iha, nfaE	iha	iha, nfaE	iha	iha	iha	iha
	**Fimbriae**	prfB	prfB	lpfA	prfB	prfB	prfB	prfB	prfB	prfB	prfB
	**secretion system**									espA_C_rod entium	
	**Autotransporter**		vat							pic, vat	vat
	**Toxins**		sat, senB	mcmA	sat	astA, sat, senB	sat	cnf1, sat, senB	sat	cba, cma, mchB, mchC, mchF, mcmA, sat	sat, senB
	**miscellaneous**	hemL	hemL	hemL, intI1, ireA	intI1, iss	iss	intI1, iss	iss	intI1	hemL, ireA, iroN	hemL

	**strain**	**DSM 101124**	**DSM 101126**	**DSM 101133**	**DSM 101134**	**DSM 101139**
**Serogenotype**	**O-Serotype**					
	**H-Serotype**	fliC H15	fliC H9	fliC H18	fliC H5	fliC H6
**Resistance associated genes against**	**Aminoglycoside -antibiotics**	aadA1, aphA, strB	aadA1	aadA1, aadB, ant2, strA, strB		aac6, aac6Ib, aadA4
	**β-lactam-antibiotics**	blaCTX-M1, blaCTX-M15, blaOXA-2	blaCTX-M1, blaCTX-M15	blaCTX-M1, blaCTX-M15, blaTEM	blaTEM	blaCTX-M1, blaCTX-M15, blaOXA-1
	**Chloramphenicol**			catA1, floR		catA1, catB3
	**Macrolid-antibiotics**	mphA, mrx			mphA, mrx	mphA, mrx
	**Tetracyclines**	tetA		tetA	tetB	tetA, tetB
	**Sulfonamide**	sul1, sul2	sul1, sul2	sul1, sul2		sul1
	**Trimethoprim**	dfrA1	dfrA1		dfrA1	dfrA17
**genes encoding virulence factors**	**Adhesins**				iha	
	**Fimbriae**		lpfA		prfB	
	**secretion system**					
	**Autotransporter**		tsh	espP	vat	
	**Toxins**		ccl, mchF	astA, toxB	sat, senB	
	**miscellaneous**	hemL, intI1	hemL, intI1, iroN, iss	hemL, intI1, iss	hemL, intI2, ireA	hemL, iss

Table S3: Phages used in this study

phage	strain for isolation	strain for propagation	origin	geographical origin	time of isolation
G28	E28	K12	manure	Hameln, Germany	Aug 2016
AB27	E53	ECOR-47	manure	Hameln, Germany	Jan 2015
KRA2	DH5α	MG1655	surface water	Portici, Italy	Apr 2008
EW2	DH5α	MG1655	surface water	Hamm, Germany	Aug 2013
TB49	E64	ECOR-13	manure	Hameln, Germany	Aug 2014
TriM	ECOR-17	ECOR-28	manure	Hameln, Germany	Mar 2016
T4 (DSM 4505)		DSM 613			

Table S4: Contents of antibiotic discs used for susceptibility testing

substance	disc content
penicillin G	10 IE
oxacillin	5 μg
ampicillin	10 μg
ticarcillin	75 μg
mezlocillin	30 μg
cefalotin	30 μg
cefazolin	30 μg
cefotaxime	30 μg
imipenem	10 μg
tetracycline	30 μg
chloramphenicol	30 μg
gentamicin	10 μg
amikacin	30 μg
vancomycin	30 μg
aztreonam	30 μg
erythromycin	15 μg
lincomycin	15 μg
ofloxacin	5 μg
norfloxacin	10 μg
colistin	10 μg
pipemidic acid	20 μg
nitrofurantoin	100 μg
bacitracin	10 IE
polymyxin B	300 IE
kanamycin	30 μg
neomycin	30 μg
doxycycline	30 μg
ceftriaxone	30 μg
clindamycin	10 μg
fosfomycin	50 μg
moxifloxacin	5 μg
linezolid	30 μg
nystatin	100 IE
teicoplanin	30 μg
quinupristin/dalfopristin	15 μg
piperacillin-tazobactam	40 μg

Table S5: Mean dimensions of phage particles with SD

phage	head width [nm]	head length [nm]	tail length [nm]
EW2	78 ± 3	80 ± 3	114 ± 6
AB27	87 ± 1	94 ± 4	96 ± 3
TB49	84 ± 3	114 ± 3	107 ± 3
TriM	49 ± 1	108 ± 1	94 ± 1
KRA2	84 ± 3	95 ± 3	109 ± 3
G28	89 ± 5	118 ± 5	107 ± 3

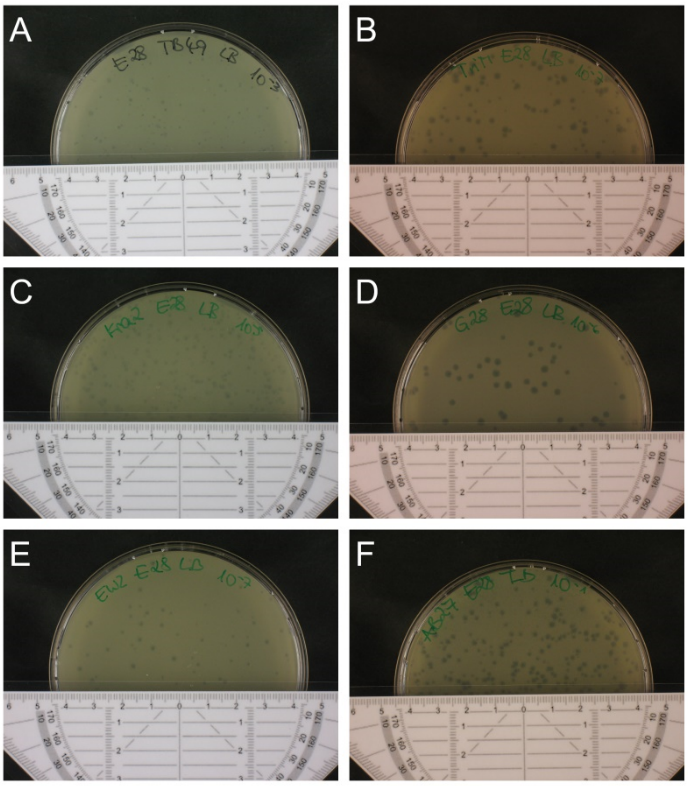

Figure S1: Plaque morphology of coliphages on E28. A) TB49, B) TriM, C) KRA2, D) G28, E) EW2 and F) AB27

Table S6: The percentage amount of strains lysed by each phage

	phages						total
	TB49	G28	KRA2	EW2	AB27	TriM	
**% of lysed strains**	50	29	21	15	18	12	67
**% of lysed clinical isolates**	53	33	8	5	15	3	70
**% of lysed isolates from poultry**	20	20	30	30	10	20	30
**% of lysed APEC strains**	54	8	38	15	15	38	77

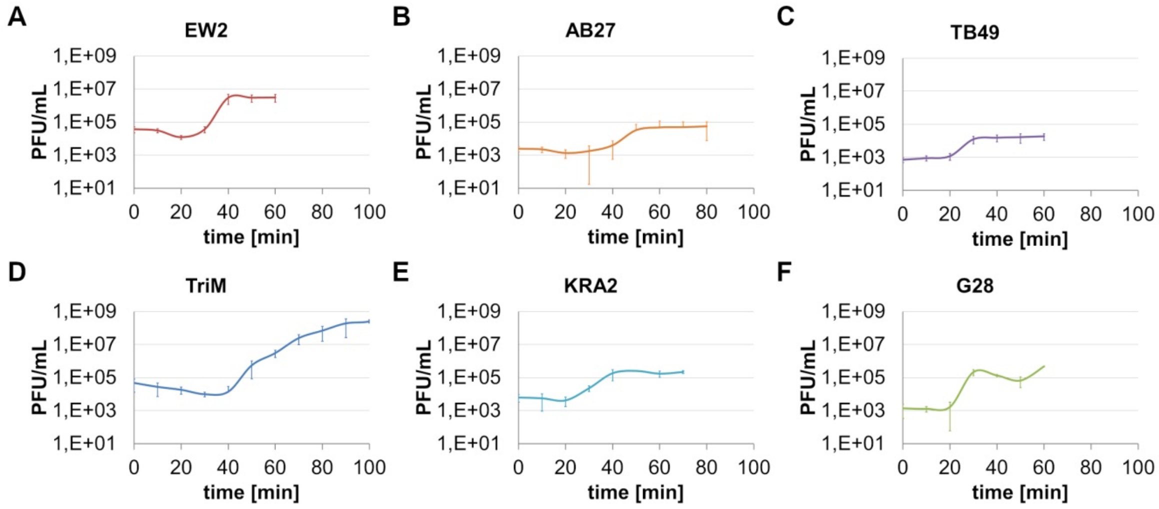

Figure S2: Growth of coliphages. One-step growth experiments of phage EW2 (A), AB27 (B), TB49 (C), TriM (D), KRA2 (E) and G28 (F) using *E. coli* E28 as host strain. Each experiment was performed three times with duplicate determinations. Error bars represent standard deviation of the mean.

Table S7: Growth of coliphages.

phage	10°C	20°C	42°C	microaerophilic	average burst size [new phage particles/cell]	latent period [min]
EW2	np	np	2.0E+00	1.9E+00	46	25
AB27	np	1.3E-02	5.1E+00	5.7E-01	6	35
TB49	np	6.0E+00	3.1E-01	6.0E+00	7	20
TriM	np	np	1.0E+00	1.1E+00	190	40
KRA2	np	5.5E-03	5.0E+00	2.0E+00	32	20
G28	1,5E-02	4.0E-01	1.3E+00	1.7E+00	80	20
Cocktail	np	1.6E-03	1.4E+00	6.3E+00		

Efficiency of plating of individual phages and the combination of the six phages (cocktail) under different growth conditions in comparison to standard conditions (37°C, aerobic, EOP = 1) and summary of burst sizes and latent periods (np = no plaques).

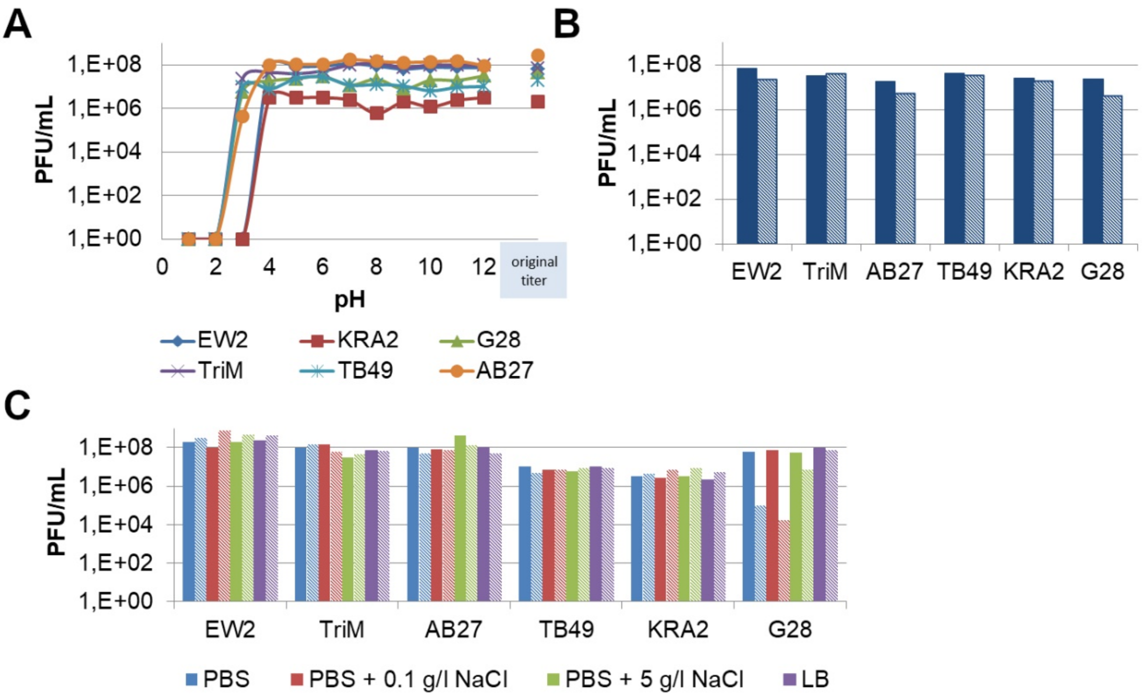

Figure S3: Stability of phages stored under different conditions. (A) The pH stability of phages after 1 h at 37°C in LB medium; (B) The titer of phages at time zero (filled bars) and after 24 hours (dashed bars) at 20°C in LB medium (pH 7); (C) Titer of phages at time zero (filled bars) and after 6 weeks (dashed bars) at 6°C in PBS buffer supplemented with different NaCl concentrations and LB medium. The means of duplicate determinations are shown.

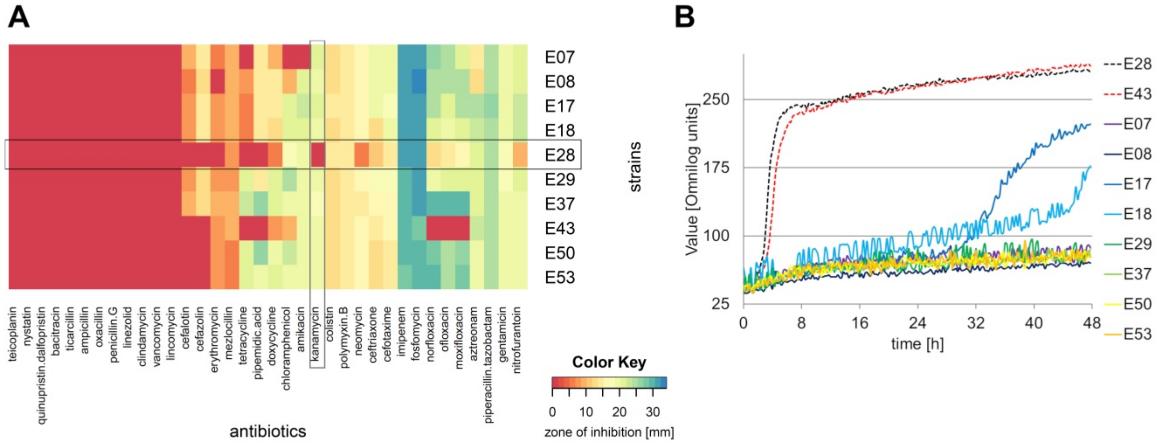

Figure S4: Antibiotic resistance patterns and resistance against potassium tellurite of 10 ESBL-producing *E. coli* isolates. (A) The susceptibility of ESBL-producing *E. coli* isolates to 36 different antibiotics is displayed as a heatmap. The color code of the heatmap is given in the bottom right corner. (B) Metabolic activity in the presence of potassium tellurite was measured using Gen III Microplates. For complete results see **Figure S1**.

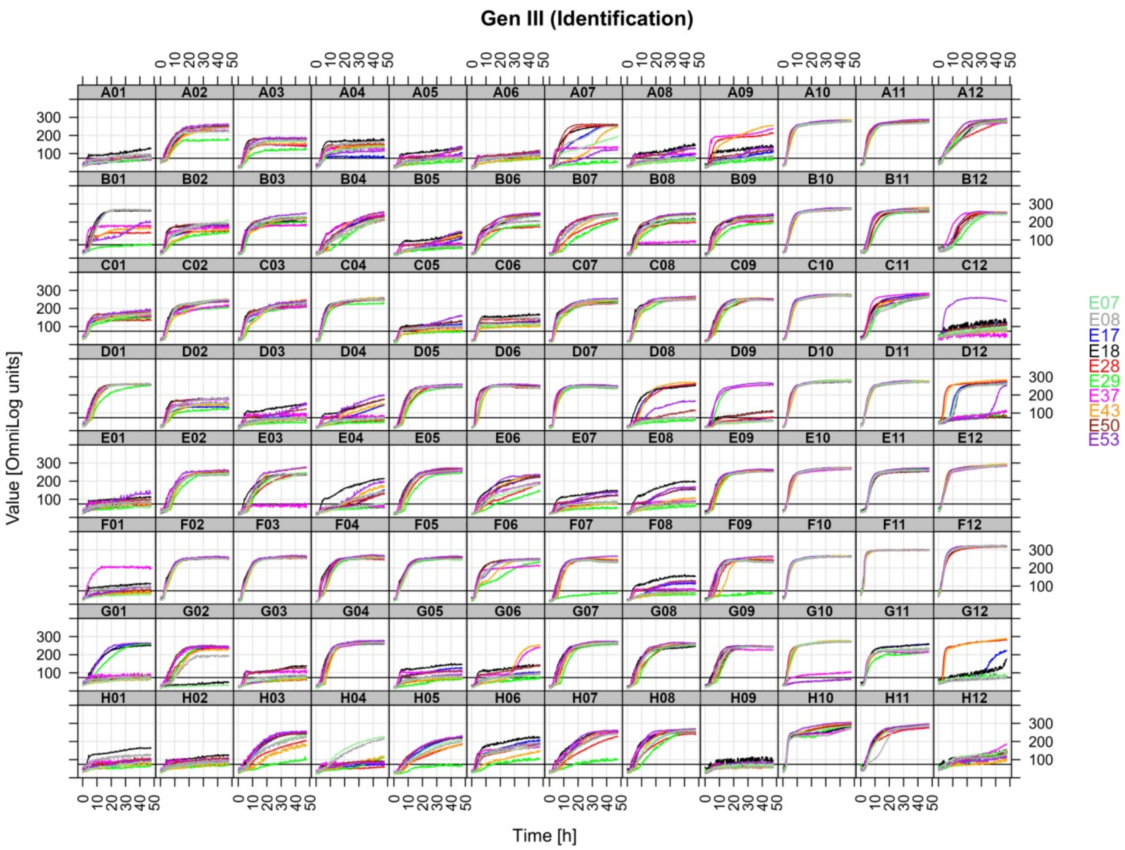

Figure S5: Results of the phenotypic array using Gen III Microplates. A01 (Negative Control), A02 (Dextrin), A03 (D-Maltose), A04 (D-Trehalose), A05 (D-Cellobiose), A06 (Gentiobiose), A07 (Sucrose), A08 (Turanose), A09 (Stachyose), A10 (Positive Control), A11 (pH 6), A12 (pH 5), B01 (D-Raffinose), B02 (α-D-Lactose), B03 (D-Melibiose), B04 (β-Methyl-D-Glucoside), B05 (D-Salicin), B06 (N-Acetyl-D-Glucosamine), B07 (N-Acetyl-β-D-Mannosamine), B08 (N-Acetyl-D-Galactosamine), B09 (N-Acetyl-Neuraminic Acid), B10 (1% NaCl), B11 (4% NaCl), B12 (8% NaCl), C01 (α-D-Glucose), C02 (D-Mannose), C03 (D-Fructose), C04 (D-Galactose), C05 (3-Methyl Glucose), C06 (D-Fucose), C07 (L-Fucose), C08 (L-Rhamnose), C09 (Inosine), C10 (1% Sodium Lactate), C11 (Fusidic Acid), C12 (D-Serine 2), D01 (D-Sorbitol), D02 (D-Mannitol), D03 (D-Arabitol), D04 (myo-Inositol), D05 (Glycerol), D06 (D-Glucose-6-Phosphate), D07 (D-Fructose-6-Phosphate), D08 (D-Aspartic Acid), D09 (D-Serine 1), D10 (Troleandomycin), D11 (Rifamycin SV), D12 (Minocycline), E01 (Gelatin), E02 (Glycyl-L-Proline), E03 (L-Alanine), E04 (L-Arginine), E05 (L-Aspartic Acid), E06 (L-Glutamic Acid), E07 (L-Histidine), E08 (L-Pyroglutamic Acid), E09 (L-Serine), E10 (Lincomycin), E11 (Guanidine Hydrochloride), E12 (Niaproof 4), F01 (Pectin), F02 (D-Galacturonic Acid), F03 (L-Galactonic Acid -Lactone), F04 (D-Gluconic Acid), F05 (D-Glucuronic Acid), F06 (Glucuronamide), F07 (Mucic Acid), F08 (Quinic Acid), F09 (D-Saccharic Acid), F10 (Vancomycin), F11 (Tetrazolium Violet), F12 (Tetrazolium Blue), G01 (p-Hydroxy-Phenylacetic Acid), G02 (Methyl Pyruvate), G03 (D-Lactic Acid Methyl Ester), G04 (L-Lactic Acid), G05 (Citric Acid), G06 (α-Keto-Glutaric Acid), G07 (D-Malic Acid), G08 (L-Malic Acid), G09 (Bromo-Succinic Acid), G10 (Nalidixic Acid), G11 (Lithium Chloride), G12 (Potassium Tellurite), H01 (Tween 40), H02 (γ-Amino-n-Butyric Acid) H03 (α-Hydroxy-Butyric Acid), H04 (β-Hydroxy-Butyric Acid), H05 (α-Keto-Butyric Acid), H06 (Acetoacetic Acid), H07 (Propionic Acid), H08 (Acetic Acid), H09 (Formic Acid), H10 (Aztreonam), H11 (Sodium Butyrate), H12 (Sodium Bromate).

Table S8: Average SD corresponding to Fig 3 (Efficacy of bacteriophages and phage combinations in inhibiting the growth of *E. coli* E28)

	LB	E28	AB27	EW2	G28	KRA2	TB49	TriM
average SD of OD 600nm 0-24h	0,008	0,007	0,008	0,248	0,004	0,041	0,032	0,017

	LB	E28	4 φ 4E6	4 φ 4E4	4 φ 4E2	4 φ 4E0	6 φ 4E6	6 φ 4E4	6 φ 4E2	6 φ 4E0
average SD of OD 600nm 0-24h	0,008	0,007	0,009	0,008	0,015	0,125	0,003	0,003	0,004	0,055

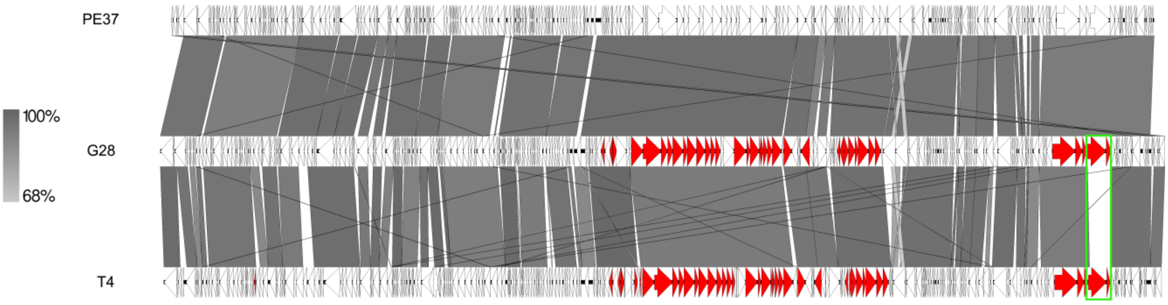

Figure S6: Genome structure of phage G28 in comparison to the related phages PE37 and T4. A synteny plot was generated using Easyfig [52] with nucleotide sequence comparison. The genome of PE37 was reversed for a better comparability. Genes for structural components are marked in red. The genes encoding a putative tail fiber tip are framed in green. The identity range is indicated by the gradient scale.

Table S9: Comparison of metabolic activity of phage-resistant variants and the wild type strain.

**Carbon source**	**E28 WT**	**E28.AB27R2**	**E28.EW2R3**	**E28.G28R3**	**E28.KRA2R3**	**E28.TB49R2c**	**E28.TriMR3**
Water	-	0	0	0	0	0	0
α-Cyclodextrin	-	0	0	0	0	0	0
Dextrin	+	0	0	-1	0	-1	0
Glycogen	-	0	0	0	0	0	0
Tween 40	-	0	0	0	0	0	0
N-Acetyl-D-galactosamine	+	0	0	-2	0	-2	0
N-Acetyl-D-glucosamine	+	0	0	-1	0	-1	0
Adonitol	-	0	0	0	0	0	0
L-Arabinose	+	0	0	-1	0	-1	0
D-Arabitol	-	0	0	0	0	0	0
D-Cellobiose	-	0	0	0	0	0	0
i-Erythritol	-	0	0	0	0	0	0
D-Fructose	+	-1	0	+1	0	+1	0
L-Fucose	+	0	0	-1	0	-1	0
D-Galactose	+	0	0	-1	0	-1	0
α-D-Glucose	+	0	0	+1	0	+1	0
m-Inositol	-	0	0	0	0	0	0
α-D-Lactose	+	0	+1	+1	0	+1	0
Lactulose	+	0	0	-2	0	-2	0
Maltose	+	0	0	-2	0	-2	0
D-Mannitol	+	0	0	+1	0	+1	0
D-Mannose	+	0	0	+1	0	+1	0
D-Melibiose	+	0	0	+1	0	+1	0
β-Methyl-D-Glucoside	+	0	0	-1	0	-1	0
D-Raffinose	+	0	0	0	0	-1	0
L-Rhamnose	+	0	0	-1	0	-1	0
D-Sorbitol	+	0	0	-1	0	-1	0
Sucrose	+	0	0	0	0	-1	0
D-Trehalose	+	-1	0	+1	0	+1	0
Xylitol	-	0	0	0	0	0	0
Methyl Pyruvate	+	0	0	-1	0	-1	0
Mono-Methyl-Succinate	+	0	0	0	0	0	0
Acetic Acid	+	0	0	-1	0	-1	0
Cis-AconiticAcid	-	0	0	0	0	0	0
Citric Acid	-	0	0	0	0	0	0
D-Galactonic Acid Lactone	-	0	0	0	0	0	0
D-Galacturonic Acid	+	0	0	-1	0	-1	0
D-Gluconic Acid	+	0	0	-1	0	-1	0
D-Glucosaminic Acid	-	0	0	0	0	0	0
D-Glucuronic Acid	+	-2	0	-1	0	-1	0
α-Hydroxy Butyric Acid	+	0	0	0	0	0	0
β-Hydroxy Butyric Acid	-	0	0	0	0	0	0
γ-Hydroxy Butyric Acid	-	0	0	0	0	0	0
p-Hydroxy Phenylacetic Acid	-	0	0	0	0	0	0
Itaconic Acid	-	0	0	0	0	0	0
α-Keto Butyric Acid	+	0	0	0	0	0	0
α-Keto Glutaric Acid	+	0	0	-2	0	-2	0
α-Keto Valeric Acid	-	0	0	0	0	0	0

D,L-Lactic Acid	+	0	0	-1	0	-1	0
Malonic Acid	-	0	0	0	0	0	0
Propionic Acid	+	0	0	-2	0	-2	0
Quinic Acid	-	0	0	0	0	0	0
D-Saccharic Acid	+	0	0	-2	0	-2	0
Sebacic Acid	-	0	0	0	0	0	0
Succinic Acid	+	0	0	-1	0	-1	0
Bromo Succinic Acid	+	0	0	-1	0	-1	0
Succinamic Acid	-	0	0	0	0	0	0
Glucuronamide	+	-2	0	0	0	-1	0
D-Alanine	+	0	0	-2	0	-2	0
L-Alanine	+	0	0	-2	0	-2	0
L-Alanylglycine	+	0	0	-2	0	-2	0
L-Asparagine	+	0	0	-1	0	-1	0
L-Aspartic Acid	+	0	0	-1	0	-1	0
L-GlutamicAcid	-	0	0	0	0	0	0
Glycyl-L-AsparticAcid	+	0	0	-2	0	-2	0
Glycyl-L-GlutamicAcid	+	0	0	-2	0	-2	0
Hydroxy-L- proline	-	0	0	0	0	0	0
L-Leucine	-	0	0	0	0	0	0
L-Phenylalanine	-	0	0	0	0	0	0
L-Proline	+	0	0	+1	0	+1	0
L-Pyroglutamic Acid	-	0	0	0	0	0	0
D-Serine	-	0	0	0	0	0	0
L-Serine	+	0	0	-1	0	-1	0
D,L-Carnitine	-	0	0	0	0	0	0
γ-Amino ButyricAcid	-	0	0	0	0	0	0
Urocanic Acid	-	0	0	0	0	0	0
Inosine	+	0	0	-1	0	-1	0
Uridine	+	0	0	-1	0	-1	0
Thymidine	+	0	0	-1	0	-1	0
Phenyethylamine	-	0	0	0	0	0	0
Putrescine	-	0	0	0	0	0	0
0-Aminoethanol	-	0	0	0	0	0	0
0,3-Butanediol	-	0	0	0	0	0	0
Glycerol	+	0	0	-1	0	-1	0
D,L-α-Glycerol Phosphate	+	0	0	-2	0	-2	0
Glucose--1- Phosphate	+	0	0	-1	0	-1	0
Glucose-6- Phosphate	+	0	0	-1	0	-1	0

Metabolic activity of phage-resistant variants was compared with that of *E. coli* E28 wild type (WT) for different carbon sources. Thereby "-" indicates no metabolic activity and "+" metabolic activity of E28 WT. Metabolic activity of phage-resistant variants was classed as follows: same as E28 WT "0", lower than E28 WT "-1", absent (in cases where E28 showed metabolic activity) "-2", higher than E28 WT "+1".

Supplemental methods:

2.2. Phage isolation, purification and propagation

After centrifugation, samples were filtrated (membrane syringe filter 0.45 µm, Sartorius, Germany), mixed with equal amounts of double concentrated LB broth and 1/20 volume of the logarithmic growing host strain. After incubation at 37 °C overnight, cells were sedimented by centrifugation at 8000 rpm (Biofuge primo R with rotor 7588 (Thermo Fisher Scientific, Waltham, Massachusetts, USA)) and the supernatant was filtrated. To isolate phages, appropriate dilutions of the enrichment were mixed with soft-agar (2.5% Miller's LB Broth Base™ powder (Invitrogen, Thermo Fisher Scientific, Waltham, Massachusetts, USA) 0.3% agar bacteriological No. 1 (w/v) (OXOID™, Thermo Fisher Scientific, Waltham, Massachusetts, USA) containing the corresponding host and overlaid on an agar plate. After incubation at 37 °C for 12-18 h, single plaques were suspended in SM buffer (100 mM NaCl, 8 mM $MgSO_4$, 50 mM Tris-HCl, pH 7.5 (Merck, Darmstadt, Germany)) using a pipette tip, followed by streaking out of the suspension on a double layer agar plate. At least four consecutive single plaque isolations were performed in order to generate a pure phage preparation, which was used for further propagation. The first lysate was produced using the isolation strain. For further production of all lysates we selected different strains for all phages. The optimal production strain for each phage was defined as the strain, with which the highest titer could be achieved (**Table S3**). In general, exponentially growing cultures were infected with phages at MOI (multiplicity of infection) 0.1, incubated for 10 min at room temperature and then at 37 °C with agitation at 123 rpm until lysis was completed or, in the absence of observable lysis, for 5 h. After centrifugation and filtration, the titer of the lysate was determined using the agar overlay method using E28 as host strain. The lysates were stored at 10 °C.

2.3. Morphology of phages and analysis of phage bacteria interactions

To analyze phage morphology, phages were allowed to adsorb onto thin carbon support films, which were prepared by sublimation of a carbon thread onto a freshly cleaved mica surface. Phages were negatively stained with 2% (w/v) aqueous uranyl acetate, pH 5.0. Samples were examined in a Zeiss EM 910 or Zeiss Libra120 Plus transmission electron microscope (Carl Zeiss, Oberkochen, Germany) at an acceleration voltage of 80 kV/120 kV and images were recorded digitally with a Slow-Scan CCD-Camera (1K ProScan/ 2K Sharp eye) with ITEM-Software (Olympus Soft Imaging Solutions, Münster, Germany). Size determination was performed from 6-10 different phages.

For FESEM bacteria and phages were fixed with 2% glutaraldehyde and 5% formaldehyde after incubation at MOI 5. Cells were centrifuged and the pellet was washed with TE-buffer (20 mM Tris, 1 mM EDTA, pH 6.9 (Merck, Darmstadt Germany), placed onto poly-L-lysine coated cover slips (12 mm in diameter) and dehydrated in a graded series of acetone (10, 30, 50, 70, 90, 100%) on ice for 15 min for each step, critical-point dried with liquid CO_2 (CPD 30, Bal-Tec, Balzers Liechtenstein) and covered with a gold-palladium film by sputter coating (SCD 500, Bal-Tec, Balzers Liechtenstein) before being examined in a field emission scanning electron microscope Merlin (Carl Zeiss, Oberkochen, Germany) using the Everhart Thornley SE detector and the inlens detector in a 25:75 ratio at an acceleration voltage of 5 kV.

The bacteriophage adsorption assay was performed as follows: *E. coli* cells (E28 and E28.G28R3) were grown in LB medium at 37 °C and agitation at 123 rpm for 2 h. 20 mL culture with a cell density of $2x10^8$ CFU/mL was prepared and phage G28 was added at a MOI of 0.1, aliquoted in 1.5 mL tubes and incubated at 37 °C in a Thermomix (Eppendorf, Hamburg, Germany). At indicated time points after phage addition an aliquot was taken, centrifuged at 8000 rpm (Biofuge primo R with rotor 7588 (Thermo Fisher Scientific, Waltham, Massachusetts, USA)) and the supernatant directly diluted in LB medium. The number of free phage particles was determined by double agar overlay with E28. Equal amounts of LB medium and phages without bacterial cells treated in the same way were used as a

control. Adsorption assays were performed three times independently and data points represent the mean and SD of triplicate measurements.

2.5. One-step growth assays

Exponentially growing cells of E28 were adjusted to $2x10^8$ CFU/mL with LB medium and infected with one of the phages EW2, AB27, TB49, TriM, KRA2 or G28 at MOI of 0.001 to ensure that a single bacterium is infected by only one phage particle. After adsorption for 10 min at room temperature cultures were centrifuged, the bacterial pellet was suspended in the initial volume of fresh LB medium and incubated at 37 °C for up to 100 min. This was done to avoid adsorption of residual free phages. At indicated time points, plaque assays were performed to enumerate viable phages in duplicate determinations. The latent period was measured as the time interval between the end of adsorption and the initial rise in phage titer. The average burst size was calculated by subtracting the titer at the end of the latent period from the maximum after the burst, divided by the number of infected bacterial cells. Data are presented as mean with standard deviation (SD) of three independent experiments.

2.9. *In vitro* biofilm model

Overnight cultures of *E. coli*, grown in LB, were adjusted to an optical density at 600 nm of 0.1 and then diluted 1:10 in fresh LB. All biofilm experiments were done in 96-well polystyrene microplates. The phage preparation used consisted of EW2, AB27, TB49, TriM, KRA2 and G28 mixed in equal parts at a concentration of $1x10^6$ PFU/mL. Wells were filled 190 µL of the bacterial culture and 10 µL of the phage preparation. Wells serving as positive controls, were filled with 190 µL of the adjusted bacterial culture and 10 µL LB, wells that served as negative controls were filled with 200 µL LB only. The microplates were then incubated for 48 h at 37 °C and 120 rpm shaking.

To study the effect of the bacteriophage preparation on already established biofilms, the latter were grown for 24 h at 37 °C and 120 rpm shaking. Then all wells were washed twice with PBS to remove non-adhered bacteria. The experimental wells were then refilled with 190 µL LB and 10 µL of the phage preparation described above. The microplates were then incubated for a second 24 h at 37 °C and 120 rpm shaking.

After 48 h, all wells were washed twice with PBS and then refilled with 200 µL of PBS. To determine viable cells, the biofilms were mechanically resuspended using a pipette tip and analysed on agar plates.

2.13. DNA isolation from phages

300 mL high titer lysate (10^{10} PFU/mL) (see 2.3.) was precipitated with 10 % polyethylene glycol 8000 and 1 M sodium chloride (both Merck, Darmstadt, Germany) followed by centrifugation for 30 min at 10000 rpm (Sorvall RC 6 Plus with rotor F10-6x500y, Thermo Fisher Scientific, Waltham, Massachusetts, USA). The pellet was resuspended in 2- 4 mL SM-buffer and treated with 10fold reaction buffer (100 mM Tris-HCl (pH 7.5), 25 mM $MgCl_2$ und 1 mM $CaCl_2$), 0.2 mg/mL RNase A and 0.002 U/µL DNase I (all Thermo Fisher Scientific, Waltham, Massachusetts, USA) followed by incubation overnight at 37 °C and 300 rpm in a Thermomix (Eppendorf, Germany). DNA was isolated using phenol-chloroform extraction and precipitated by 3 M sodium acetate and 100% ethanol. After incubation for 15 min at -80 °C, DNA was pelleted by centrifugation and washed twice with 70% ethanol. The pellet was air-dried and solved in 50- 200 µL TE-buffer (10 mM Tris-HCl, 1 mM EDTA, pH 8, Merck, Darmstadt Germany). The concentration was determined using the Qubit® dsDNA HS Assay Kit (Thermo Fisher Scientific, Waltham, Massachusetts, USA) following the manufacturer`s instructions.

© 2020 by the authors. Submitted for possible open access publication under the terms and conditions of the Creative Commons Attribution (CC BY) license (http://creativecommons.org/licenses/by/4.0/).
