# Supplementary figures and images for: In Vitro Evaluation of a Phage Cocktail Controlling Infections with Escherichia coli
Source: Viruses. 2020 Dec 19;12(12):1470. doi: 10.3390/v12121470 (PMC7768485; doi:10.3390/v12121470)

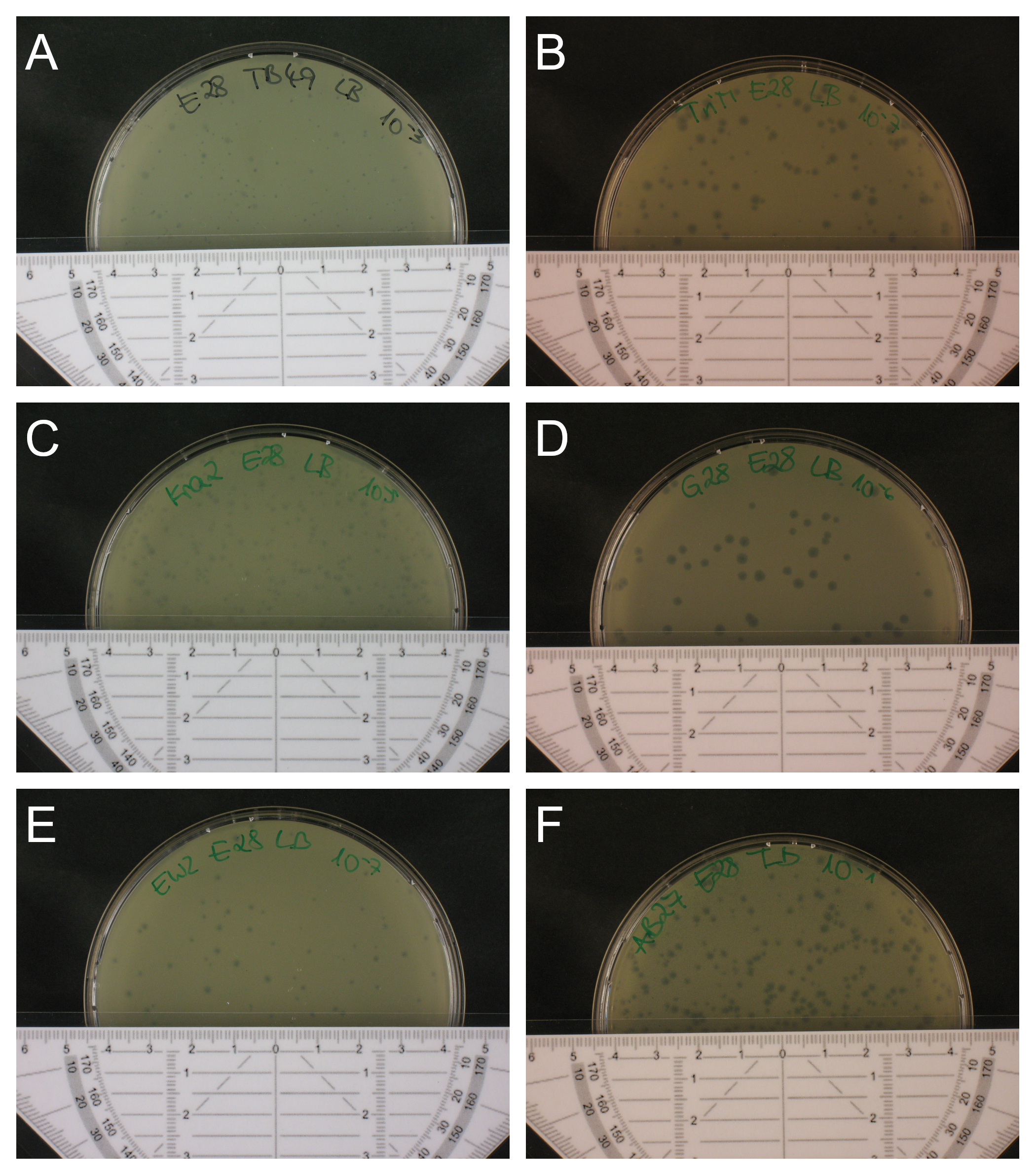

Supplement: Supplementary file 1 [file viruses-12-01470-s001.zip › viruses-974160-1/Supplements/FigureS1.jpg]

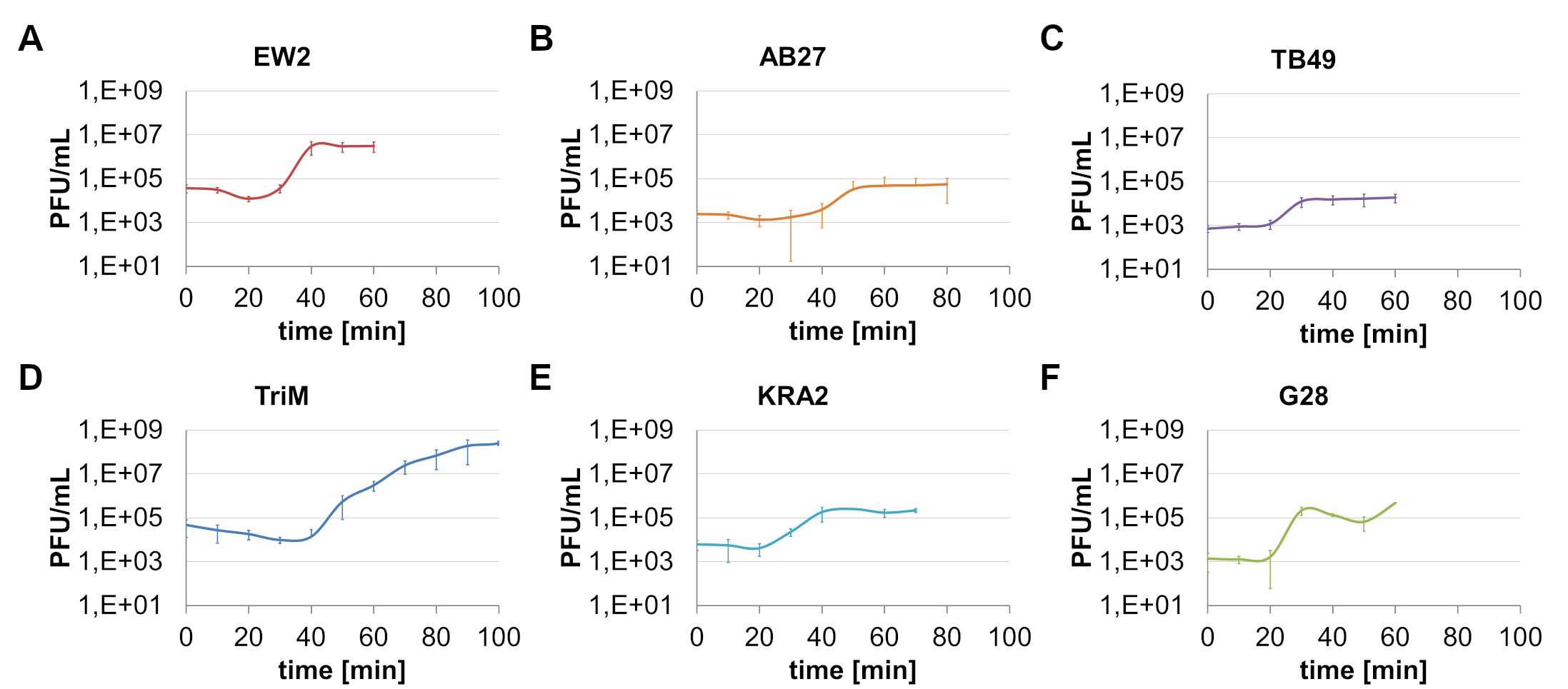

Supplement: Supplementary file 1 [file viruses-12-01470-s001.zip › viruses-974160-1/Supplements/FigureS2.jpg]

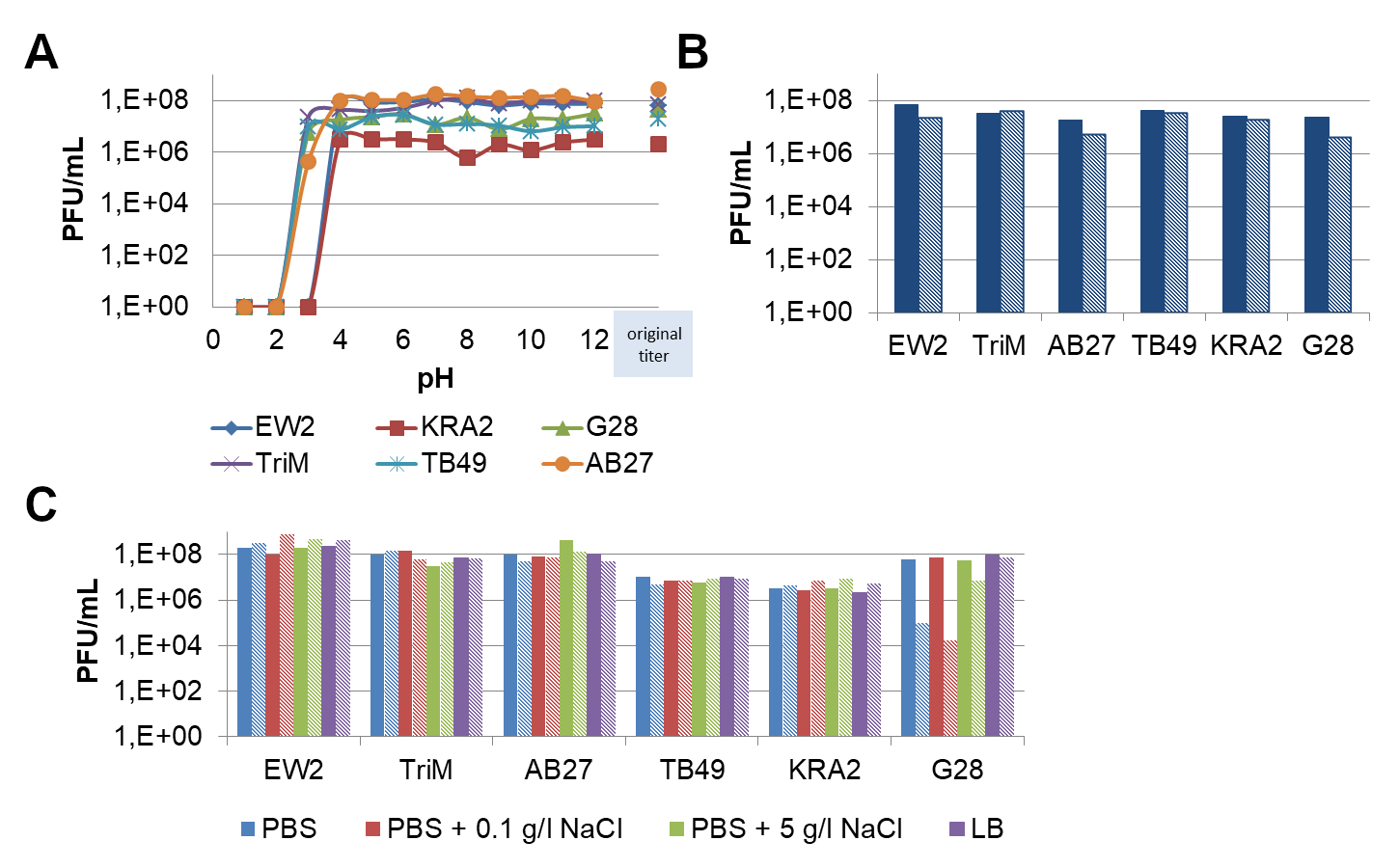

Supplement: Supplementary file 1 [file viruses-12-01470-s001.zip › viruses-974160-1/Supplements/FigureS3.jpg]

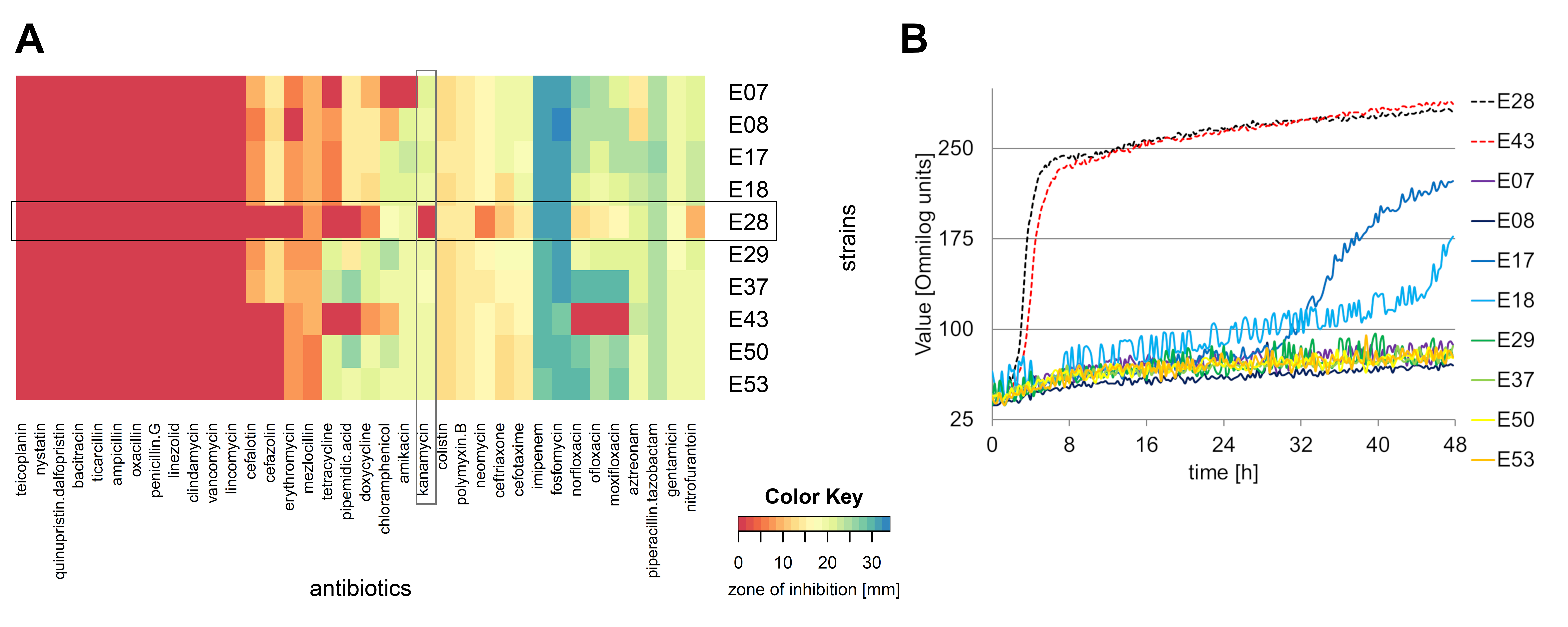

Supplement: Supplementary file 1 [file viruses-12-01470-s001.zip › viruses-974160-1/Supplements/FigureS4.jpg]

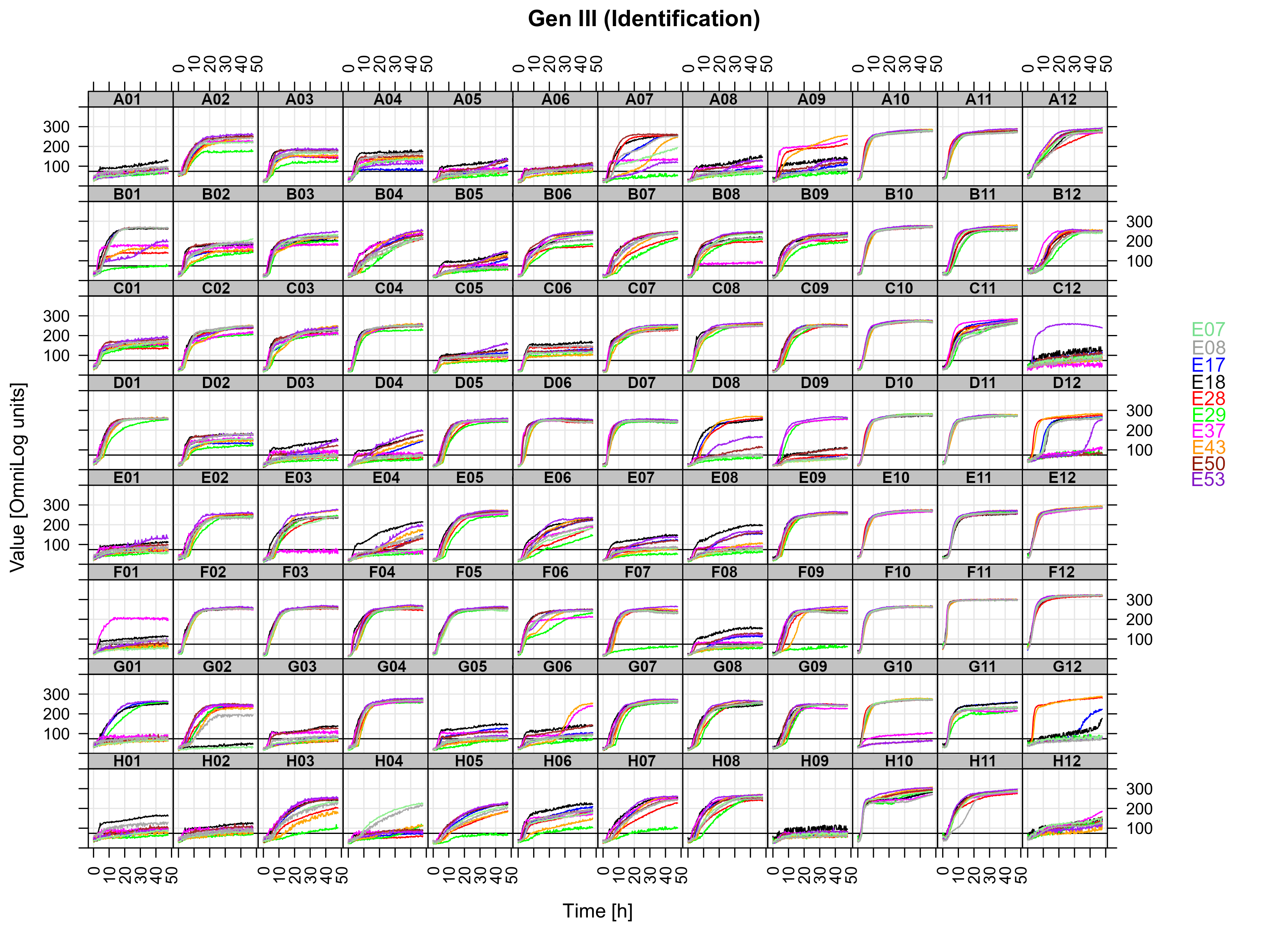

Supplement: Supplementary file 1 [file viruses-12-01470-s001.zip › viruses-974160-1/Supplements/FigureS5.jpg]

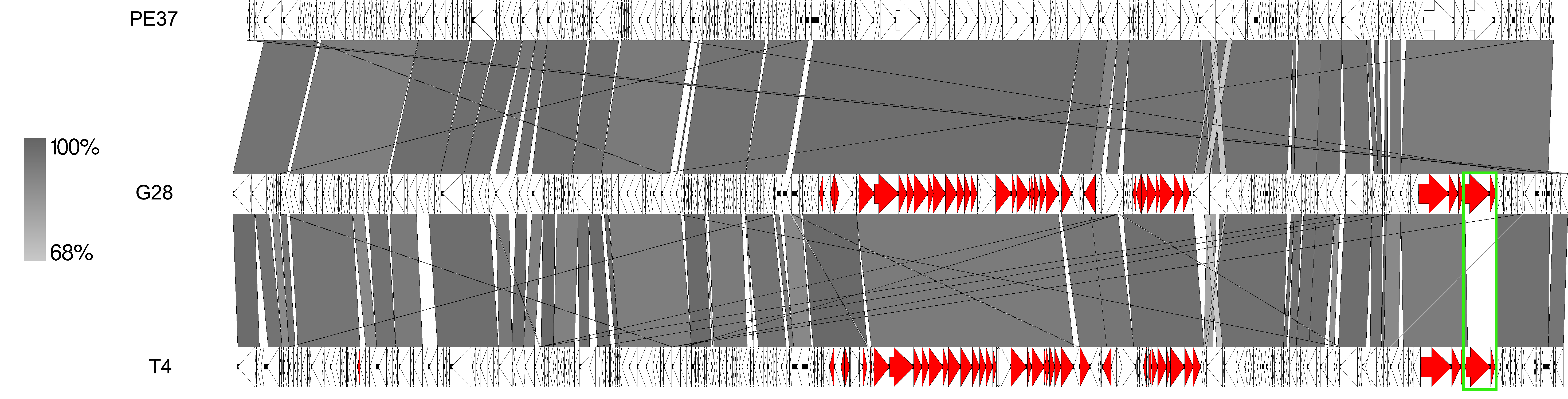

Supplement: Supplementary file 1 [file viruses-12-01470-s001.zip › viruses-974160-1/Supplements/FigureS6.tif]
